# Differentiating Thermal Conductances at Semiconductor Nanocrystal/Ligand and Ligand/Solvent Interfaces in Colloidal Suspensions

**DOI:** 10.1021/acs.nanolett.2c04627

**Published:** 2023-04-24

**Authors:** Yuxing Liang, Benjamin T. Diroll, Kae-Lin Wong, Samantha M. Harvey, Michael Wasielewski, Wee-Liat Ong, Richard D. Schaller, Jonathan A. Malen

**Affiliations:** † Department of Mechanical Engineering, Carnegie Mellon University, 5000 Forbes Ave., Pittsburgh, Pennsylvania 15213, United States; ‡ Center for Nanoscale Materials, Argonne National Laboratory, 9700 S Cass Ave., Lemont, Illinois 60439, United States; § ZJU-UIUC Institute, College of Energy Engineering, Zhejiang University, 718 East Haizhou Road, Hangzhou 310058, People’s Republic of China; △ Department of Chemistry, Northwestern University, Evanston, IL 60208, United States

**Keywords:** semiconductor nanocrystal, Interfacial thermal conductance

## Abstract

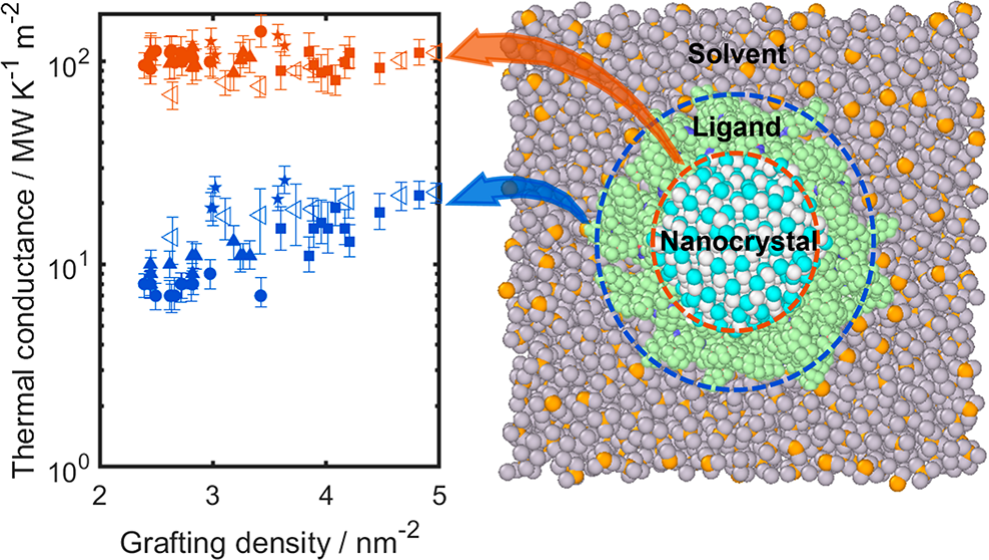

Infrared-pump, electronic-probe (IPEP) spectroscopy is used to measure heat flow into and out of CdSe nanocrystals suspended in an organic solvent, where the surface ligands are initially excited with an infrared pump pulse. Subsequently, the heat is transferred from the excited ligands to the nanocrystals and in parallel to the solvent. Parallel heat transfer in opposite directions uniquely enables us to differentiate the thermal conductances at the nanocrystal/ligand and ligand/solvent interfaces. Using a novel solution to the heat diffusion equation, we fit the IPEP data to find that the nanocrystal/ligand conductances range from 88 to 135 MW m^–2^ K^–1^ and are approximately 1 order of magnitude higher than the ligand/solvent conductances, which range from 7 to 26 MW m^–2^ K^–1^. Transient nonequilibrium molecular dynamics (MD) simulations of nanocrystal suspensions agree with IPEP data and show that ligands bound to the nanocrystal by bidentate bonds have more than twice the per-ligand conductance as those bound by monodentate bonds.

The practicality of semiconductor nanocrystal-based technologies, including light-emitting diodes,^1,2^ lasers,^3^ thermal therapies,^4−6^ and thermoelectric generators,^7^ depends in large part on thermal transport. Organic ligands help stabilize the nanocrystals during the solution-based growth process but act as electrical and thermal barriers in practical applications. Semiconductor nanocrystals, of which CdSe are the most studied, have attracted interest due to their size-dependent properties (e.g., band gap) and convenient processing.^8,9^ In various compositions, semiconductor nanocrystals are promising for nanomedicine diagnostics and therapies where semiconductor nanocrystals are suspended in solvents.^6^ Whether in optoelectronics or medicine, heat transfer across the solid/solid or solid/fluid interfaces plays an important role in defining the operational temperature of the semiconductor nanocrystals, which affects performance. Thus, understanding thermal transport across the interfaces of the semiconductor nanocrystal/ligand/solvent systems is essential for designing semiconductor nanocrystals that deliver or dissipate heat efficiently. Prior measurements, however, consider metal nanocrystals, while few measurements exist for semiconductor nanocrystals suspended in solvents, and none can discern the separate conductances at the nanocrystal/ligand vs ligand/solvent interfaces.

Experimentalists have measured interfacial thermal conductance (*h*) between organic and inorganic materials, which defines the heat flux (*q*′′) per unit temperature jump (Δ*T*) across the interface . Measurements and simulations of *h* across self-assembled monolayers (SAMs) sandwiched between solids found that it scales with the SAM’s bonding strength and the vibrational alignment of the contacts.^10−14^ Others have considered *h* at solid/SAM/fluid interfaces. Wang el al.^15^ used sum-frequency generation spectroscopy to probe planar SAMs anchored to a gold substrate and exposed to air on the other side. Using femtosecond laser pulses absorbed by the molecules, they measured *h* across the metal/ligand interface to be 220 ± 100 MW m^–2^ K^–1^, which is comparable to *h* of some metal/semiconductor interfaces. Ge et al.^16^ used picosecond transient absorption to measure thermal transport across interfaces of AuPd nanocrystals suspended in water and organic solvent, respectively, and found that *h* of a metal nanocrystal/ligand/aqueous solvent system is between 100 and 300 MW m^–2^ K^–1^ while that at the metal nanocrystal/ligand/organic solvent system is only 15 MW m^–2^ K^–1^. Nguyen et al.^17^ used time-resolved infrared spectroscopy to excite suspended gold nanorods with a pump laser and then probe the absorption change of the solvent, corresponding to a change in temperature. They found that the heat transfer is inhibited by the silica coating of the nanorods.

All prior measurements only determine the combined *h* of the nanocrystal/ligand and ligand/solvent interfaces and rely on excitation schemes that limit their applicability to metallic nanocrystals. In metal nanocrystals, which have no band gap, excited electrons reach thermal equilibrium with the lattice in a few picoseconds and long-lived signals in transient absorption measurements convey the temperature of the particles. However, in direct band gap semiconductor nanocrystals, excitation of electrons across the band gap results in reduced absorption (termed bleaching), which generates an intense transient absorption signal that overwhelms thermochromic shifts in the band gap and often persists for several nanoseconds or longer.

In this work, we used infrared pump, electronic probe spectroscopy (IPEP)^18,19^ to separate the nanocrystal/ligand and ligand/solvent thermal conductances of CdSe semiconductor nanocrystals suspended in organic solvents and developed numerical and analytical models to understand the results. In IPEP measurements, an infrared pump pulse with the spectrum shown by a black line in Figure S3 selectively excites the C–H vibrations of the oleate ligands. Since the cross-section of oleate is very small (peaking at 1.8 × 10^–18^ cm^2^, as calculated in Section 1.3 of the Supporting Information), only 2% of ligands are excited by each pulse at the 3.8 mJ cm^–2^ pump fluence. This number represents an average of one to five ligands excited per nanocrystal, per pulse, depending primarily on the nanoparticle diameter. Absorption of the infrared pulse into the C–H vibrational modes of the oleate ligands leads to a cascade of heat transfer phenomena shown in Figure 1a. First, ligands themselves undergo intramolecular vibrational relaxation (IVR), which typically takes picoseconds in a dense phase such as a fluid or solid.^20,21^ IVR redistributes energy from C–H modes to all available phonon modes of the ligands, raising the temperature of the excited ligands. Second, heat is transferred in parallel from the heated ligands to nonexcited ligands, nanocrystal core, and solvent medium. There may also be direct heat transfer between the nanocrystal and the solvent. The temperature change of the nanocrystals is then determined by detecting the transient absorbance change of the NC (using ultrafast spectroscopy) that is calibrated by collecting static temperature-dependent absorption spectra at small temperature increments and differencing data (*A*_*T*_ – *A*_295 K_) as shown in Figure 1b.

**Figure 1:**
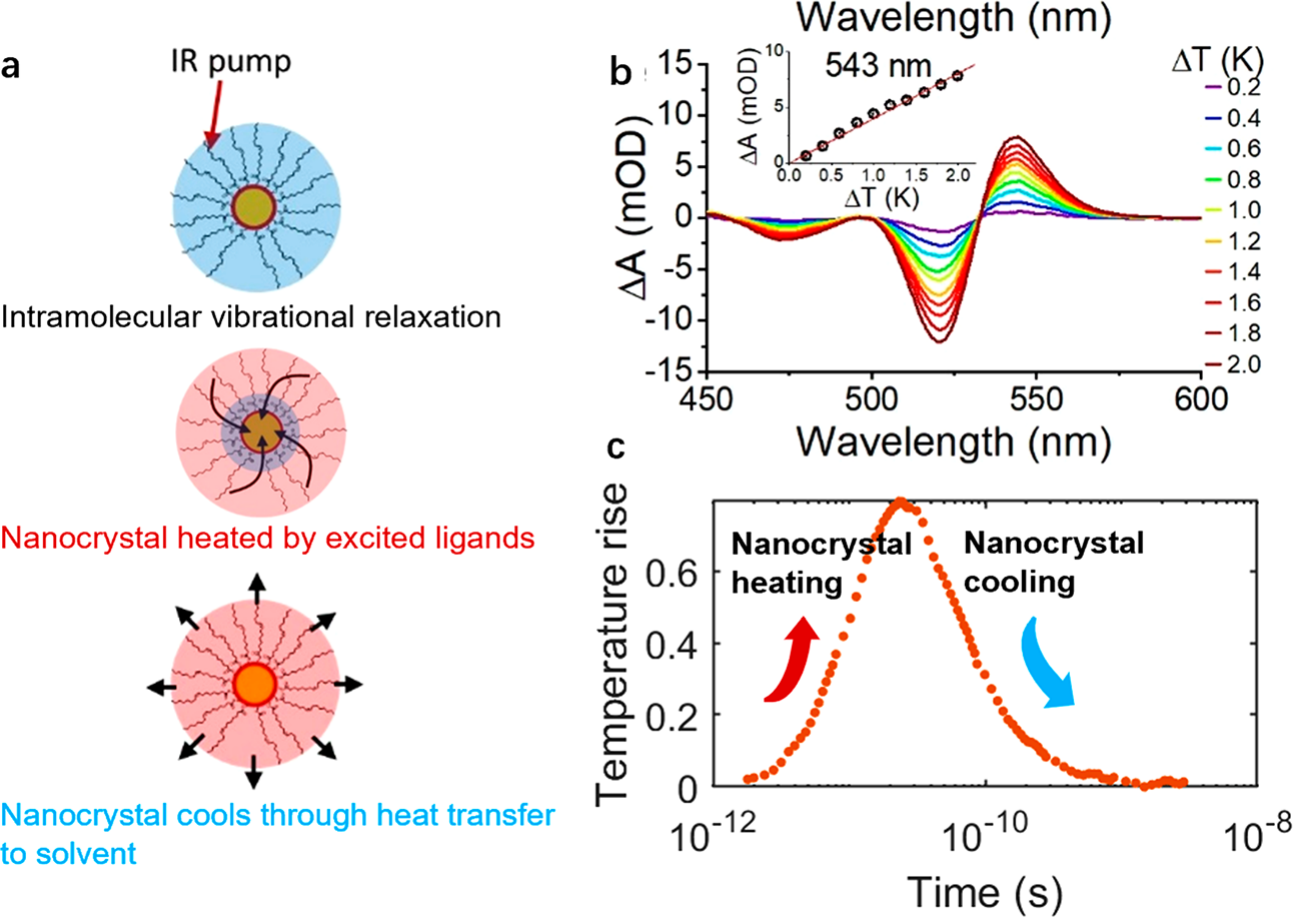
(a) Sketch of the three heat transfer processes. (b) Static state measurement and calibration of temperature change to absorption change. (c) Temperature change of IPEP measurement in the range of 0–1000 ps.

Four sizes of CdSe nanocrystals capped with oleate ligands with controlled grafting densities were synthesized and measured by IPEP. Grafting density was controlled by subjecting samples to antisolvent precipitation using different polar solvents, then measured from organic weight loss in thermogravimetric analysis experiments (Section 1.2 of the Supporting Information). The transient temperature change of the CdSe nanocrystals is exemplified in Figure 1c. The data show that there is a heating process of the particle for up to 20 ps as heat enters the CdSe nanocrystals from the excited ligands. The subsequent cooling process occurs over approximately a nanosecond before the CdSe equilibrates with the solvent. To interpret the heat transfer process, we developed a 3D finite element model and a more computationally efficient radial symmetric model that accurately reproduces the IPEP data. Through fitting the data, the radial symmetric model is used to determine *h* at the semiconductor nanocrystal/ligand/solvent interfaces. With this model, we find that the ligand/solvent interface is a bottleneck to heat transfer. Additionally, we perform molecular dynamics (MD) simulations that reveal vastly different conductances from monodentate and bidentate bonds at the nanocrystal/ligand interface.

The commercial finite element analysis (FEA) software ANSYS was used to predict the heat transfer process in the colloidal system. The schematic diagram of heat transfer in the semiconductor nanocrystal/ligand/organic solvent system is shown in Figure 2b.1. The geometry is approximated by three concentric spheres representing the CdSe nanocrystal, capped organic ligands, and surrounding solvent. ANSYS solves the heat diffusion equation
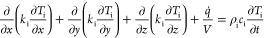
1where the subscript i refers to the CdSe, excited oleate ligands, nonexcited ligands, and CCl_4_ solvent respectively, *k* is the thermal conductivity, ρ is the density, and *c* is the corresponding specific heat capacity. The thermal properties of materials are referenced,^22−24^ and the density of the ligand is calculated by the grafting density. Interligand heat transfer is considered by assigning the ligands the thermal conductivity of pure oleate. Due to the limited cross section for excitation by photons, volumetric heat generation *q̇* takes places in selected elements of the ligand layer (shown as the red region in Figure 2b.1).

**Figure 2:**
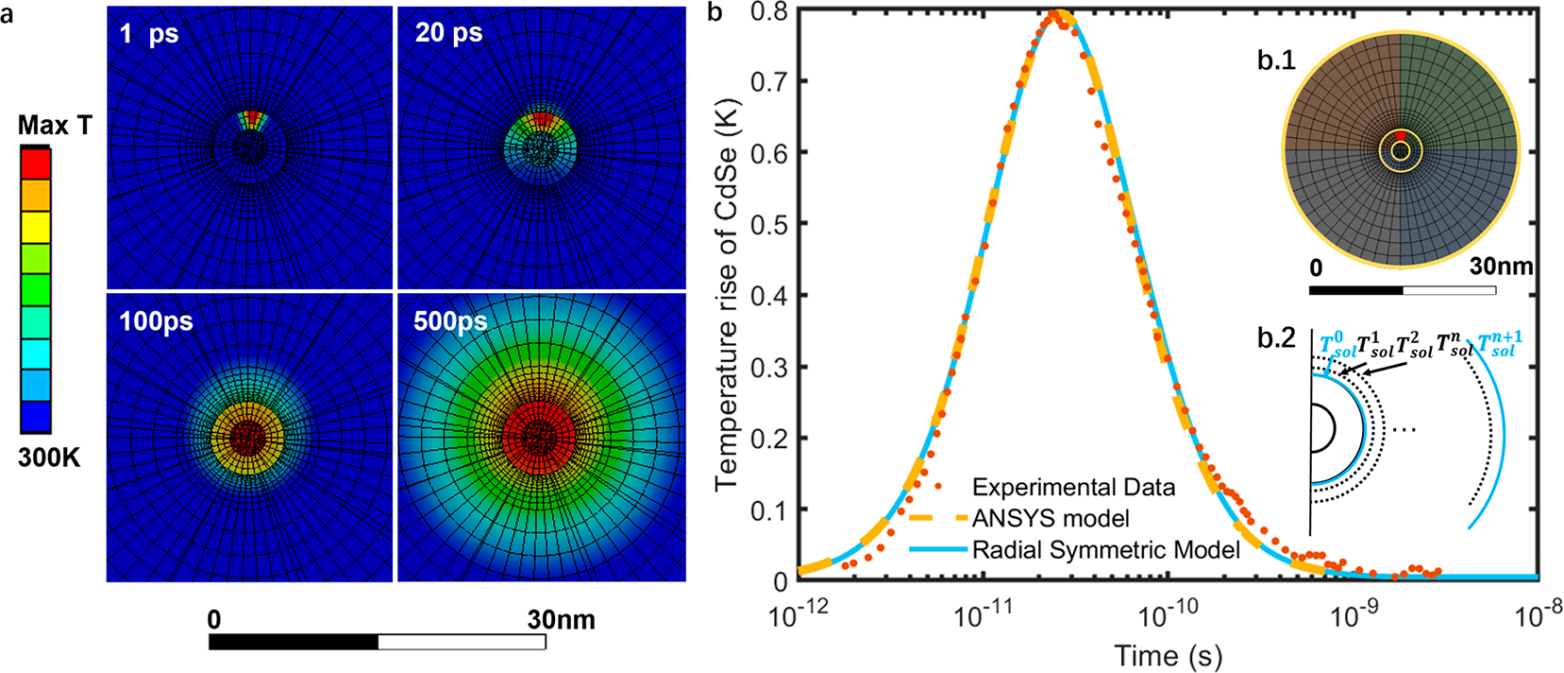
Models of IPEP process on a 4.7 nm nanocrystal. (a) Temperature profile calculated by the ANSYS model at 1, 20, 100, and 500 ps. (b) ANSYS model and the radial symmetric heat diffusion model compared with the experimental data; Inset b.1: schematic diagram of the ANSYS model, showing different mesh regions for CdSe, oleate, and solvent from inside to outside; the red area refers to the excited ligand; Inset b.2. schematic diagram of the radial symmetric model, showing the CdSe, oleate, and discretized spatial domain of the solvent.

The heat transfer process for a 4.7 nm CdSe nanocrystal with 2.8 nm^–2^ grafting density is shown in Figure 2a. Though a small region of ligands are initially excited, the heat transfer process at long time scales is radially symmetric. Heat is first generated in the excited ligands and transferred to the nonexcited ligands and into the nanocrystal. After approximately 100 ps the nanocrystal has the highest temperature in the system. Finally, the heat spreads radially from the nanocrystal/ligand system and into the solvent.

ANSYS solves the 3D differential equations and deals with the asymmetric excitation and heat transfer process. Nevertheless, it takes a large amount of computational time to complete the hundreds of simulations needed to identify the combination of *h*_lig–NC_ and *h*_lig–sol_ that best fits the data, a process that needs to be repeated for different grafting densities and nanocrystal diameters. As an example, for the model of the 4.7 nm CdSe nanocrystal with 2.8 nm^–2^ grafting density, 416 simulations with different combinations of the thermal conductances were used to adequately resolve the best fit values of *h*_lig–sol_ and *h*_NC–lig_. Inspired by the observation that heat transfer is primarily radial, we next develop an analytical model with radial symmetry to streamline subsequent fitting of multiple data sets.

In the radial symmetric heat diffusion model the geometry of the capped nanocrystal is approximated by two concentric spheres, surrounded by solvent. The inner layer represents the CdSe nanocrystal, and the outer layer represents the organic ligands. With the lumped capacitance assumption of uniform temperature within the nanocrystal and ligand layers, the temperature rise can be described by the following two ordinary differential equations (ODE) based on the energy balance of the ligand layer (eq 2) and nanocrystal layer (eq 3)
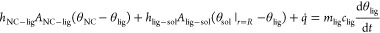
2
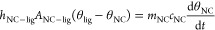
3where the subscripts NC, lig, and sol refer to the CdSe, oleate ligands, and CCl_4_ solvent, respectively, θ is the temperature increase (θ = *T*(*t*) – *T*(*t* = 0)), *A* represents the area of the interfaces, and *R* represents the radius of the interface between the ligand and the solvent . The volumetric heat generation rate, *q̇*, is described by an exponential decay with a time constant of 6 ps due to excitation of the C–H bonds and energy redistribution by IVR. The specific value of the time constant is obtained by minimizing the aggregated root-mean-square error of the fit to all the data sets and is consistent with previously reported IVR time scales.^20,21^ Interfacial conductances that dictate nanocrystal heating and cooling relate to much longer time scales (10–1000 ps) and are insensitive to the exact value of this time constant.

To describe heat transfer into the solvent, the heat diffusion equation in the radial coordinate system is
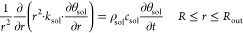
4where *R*_out_ defines the boundary of the spatial domain. The boundary condition at the ligand/solvent interface is
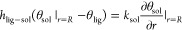
5which implies that heat flux is continuous, while a temperature jump dictated by *h*_lig–sol_ exists. At the outer boundary of the spatial domain, the heat flux is zero
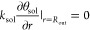
6due to a symmetrical temperature response from neighboring nanocrystals excited by the same pulse.

This partial differential equation (PDE)–ODE system cannot be solved analytically. We discretized the spatial domain of the solvent into *N* shells with the central difference method as shown in Figure 2b to convert the PDE to a nonhomogeneous ODE system in time that can be solved analytically (see Section 3.2 in the Supporting Information for details) with the initial condition that θ_lig_ = 0 and θ_NC_ = 0. The solution is then fit to the experimental data to determine *h*_NC–lig_ and *h*_lig–sol_ by minimizing the mean squared error, , where θ_*j*_ is the experimental data and θ(*t*_*j*_) is the prediction. The best fit from this model results in *h*_NC–lig_ = 105_–18_^+20^ MW m^–2^ K^–1^, *h*_lig–sol_ = 25_–2_^+2^ MW m^–2^ K^–1^ with χ = 0.02 K, which quantifies the excellent quality of the model fit. The reported uncertainty in the conductances is based on the sensitivity of the fit as described in Section 3.3 in the Supporting Information. The best fit values of *h*_lig–NC_ and *h*_lig–sol_ from this radial symmetric model are within 20% of those predicted by ANSYS for this data set. More comparison between the results from the two models can be found in Section 3.4 in the Supporting Information.

Similar results from the two models show that the assumptions made in the radial symmetric model are reasonable. The finite element model assumes that only a single localized bundle of ligands is excited, while the radially symmetric model assumes that the entire spherical shell of ligands is symmetrically heated by the pump laser. The two models consider two extreme conditions, but the fitted results are well aligned, which shows that intermediate conditions where multiple local bundles are excited would make little difference in the predictions of thermal conductance. Similarly, the finite element model defined the thermal conductivity of the ligands as that of pure oleate which allows the heat to flow circumferentially. For the radial symmetric model, the ligand shell is assumed to be uniform in temperature (i.e., infinite thermal conductivity). Again, the very similar results of the two models verify that ligand thermal conductivity has little influence on the temperature response. Hence, moving forward to all the experimental data sets on the samples with different diameters and grafting densities, we have used the radial symmetric model, instead of the finite element model, to reduce computation time.

We report the values of *h*_NC–lig_ and *h*_lig–sol_ as determined by the radial symmetric model, as a function of ligand grafting density in Figure 3a. For all data sets *h*_NC–lig_ is much larger than *h*_lig–sol_. As shown in Figure 3b, it was previously reported^16,17^ that the effective thermal conductance across the metal nanocrystal/ligand/aqueous solvent interface is between 100 and 250 MW m^–2^ K^–1^ while that at the metal nanocrystal/ligand/organic solvent interface is only 15 MW m^–2^ K^–1^. Therefore, it was hypothesized that the thermal conductance at the ligand/solvent interface is the bottleneck to heat transfer in these systems. The IPEP method and our analysis enables explicit separation of the two interfacial thermal conductances for the first time, and our examination confirms this hypothesis. As shown in Figure 3a, the nanocrystal/ligand conductance ranges from 88 to 135 MW m^–2^ K^–1^ while the ligand/solvent conductance ranges from 7 to 26 MW m^–2^ K^–1^. The average thermal conductance of the nanocrystal/ligand interface (103 MW m^–2^ K^–1^) is almost 1 order of magnitude larger than that of the ligand/solvent interface (14 MW m^–2^ K^–1^). This discrepancy reveals that the covalent bond between the ligands and the nanocrystal enables the vibrational energy transfer faster than the van der Waals interactions between the ligands and the solvent. Notably *h*_NC–lig_ values are relatively constant over the measured range of grafting density, while *h*_lig–sol_ weakly increases with grafting density.

**Figure 3:**
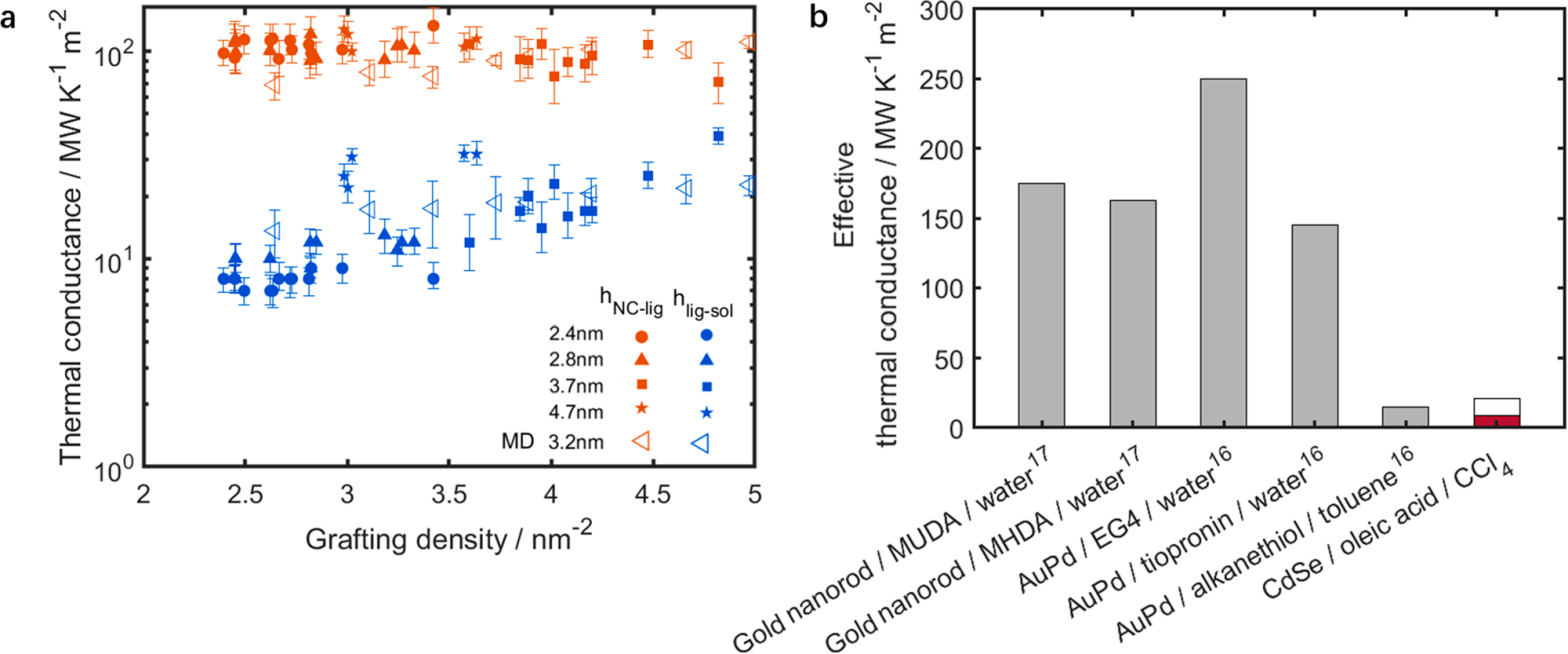
(a) Results of the thermal conductances of the CdSe nanocrystals with different sizes and grafting densities predicted by the radial symmetric model and by MD simulations. (b) Range of effective thermal conductances (shown as the open range)  from IPEP data is compared to prior studies of similar systems,^16,17^ where MUDA is mercaptoundecanoic acid, MHDA is mercaptohexadecanoic acid, and EG4 is monohydroxy(1-mercaptoundec-11-yl)tetraethylene glycol. IPEP measurements of *h*_eff_, shown in maroon, agree with those done in organic, but not aqueous, colloidal suspensions.

To understand the atomistic origins of the experimental thermal conductance trends, we used MD simulations to study a similar system consisting of a CdSe nanocrystal with oleic acid ligands immersed in a solvent of CCl_4_ molecules. For the study of thermal conductance between nanocrystal/ligand interface, both protonated and deprotonated states (oleate vs oleic acid) using MD simulations are considered and the results are indistinguishable within the error of MD. More discussion can be found in Section 4.4 in the Supporting Information. Details of the potentials^25−30^ and simulation can also be found in Section 4.2 in the Supporting Information. Briefly, a CdSe nanocrystal with a diameter of 3.2 nm was grafted with oleic acid molecules and surrounded by excess CCl_4_ molecules. The nanocrystal center was tethered by a stiff spring to the center of an 8 × 8 x 8 nm^3^ simulation cell. The resulting system was equilibrated to a pressure of 0 atm and a temperature of 290 K using an NPT ensemble. After stabilizing the system in an NVE ensemble, the interfacial thermal conductances between the nanocrystal/ligand and the ligand/solvent were calculated using a transient heat conduction method^31^ (Section 4.2 in the Supporting Information) that is consistent with the above radial symmetric model.

The average thermal conductances for the two interfaces calculated from the MD simulations are plotted in Figure 3a. The values of *h*_NC–lig_ and *h*_lig–sol_ from MD agree with the experimental data over the entire range of grafting densities. The close agreement with the experimental results suggests that reliable molecular-level insights of the thermal transport physics are captured in the MD simulations.^32^

Figure 4a–c depicts a cross-sectional view of a CdSe nanocrystal with 100 attached oleic acid ligands and surrounded by CCl_4_ molecules. The O atoms (i.e., the carbonyl oxygen O1 and hydroxyl oxygen O2) on the ligands are bonded to the Cd atoms from the nanocrystal. As shown in the inset of Figure 4, the oleic acid ligand can be attached to the nanocrystal with a monodentate (through the O1 atom) or bidentate bond. We plotted the number of monodentate and bidentate ligands at various grafting densities in Figure 4d (Section 4.3 in the Supporting Information for details). The number of total bonds between the ligands and the nanocrystal increases monotonically with the number of attached ligands. However, this increase is not shared equally between the numbers of monodentate and bidentate ligands. The monodentate-bound ligand is favored at higher grafting density due to the smaller steric influence from its smaller footprint on the nanocrystal’s surface.

**Figure 4:**
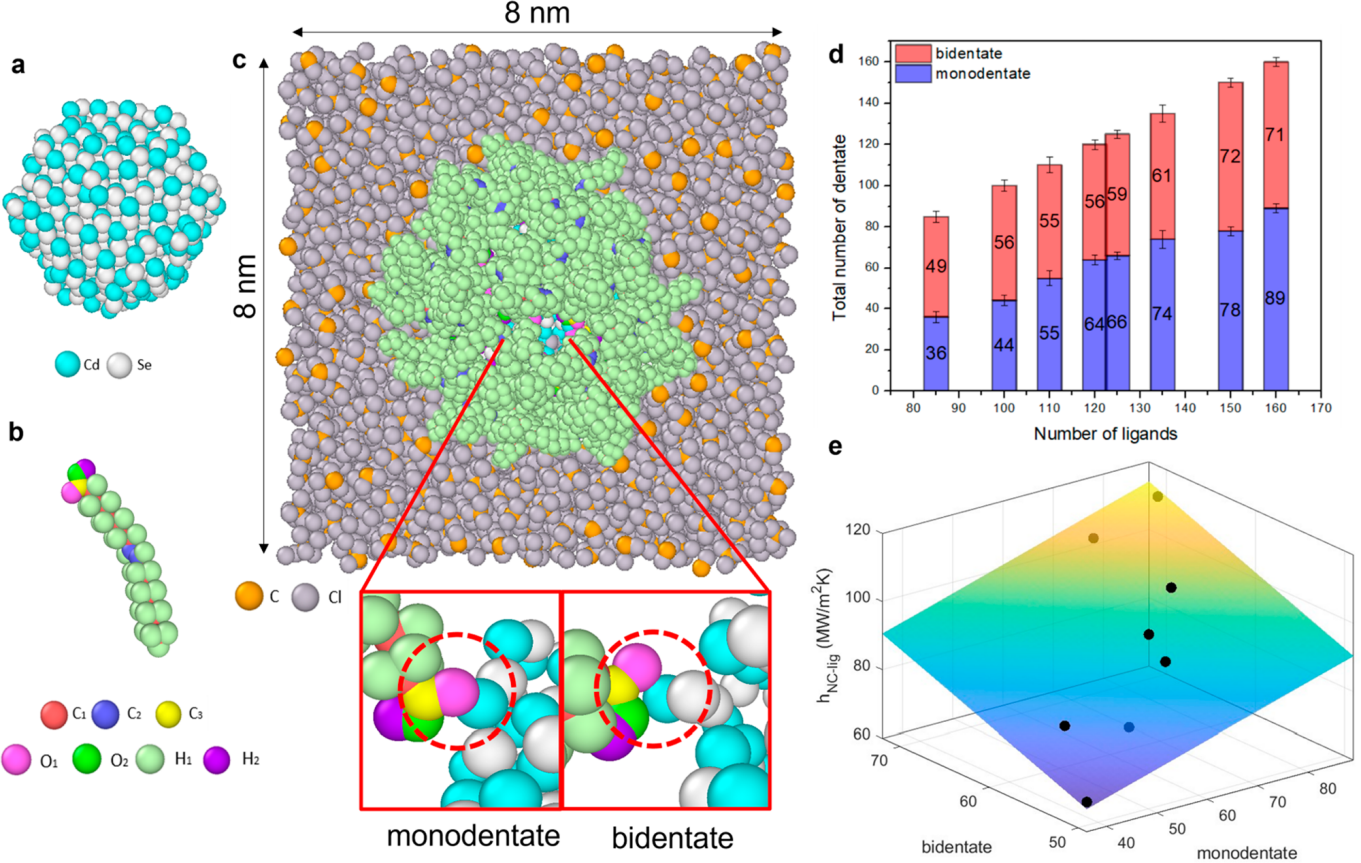
Simulation setup: (a) CdSe nanocrystal; (b) Oleic acid ligand; (c) CdSe nanocrystal with 100 oleic acid ligands and CCl_4_ solvent molecules. The diameter of the nanocrystal is about 3.2 nm, and the cubic simulation box has a length of 8 nm on each side with periodic boundary conditions applied at all three spatial directions. The inset illustrates the different types of molecular bonds between the oleate ligands and CdSe nanocrystal. (d) The total number of bonds versus the total number of ligands attached to the CdSe nanocrystal. The uncertainty bars on these plots are from counting the bonds between Cd and O atoms at five different time steps during a simulation run. (e) The fit result for the interfacial thermal conductance contributed by an individual monodentate or bidentate bond using the total interfacial thermal conductance.

The observed thermal conductance at the ligand/nanocrystal interface is, thus, influenced by the number of monodentate and bidentate ligands. We fit the simulated thermal conductance as a function of the number of monodentate and bidentate ligands to estimate the thermal conductance per type of bonding. The fit result plotted in Figure 4e gives a value of 0.45 ± 0.29 MW m^–2^ K^–1^ per monodentate ligand and 1.03 ± 0.31 MW m^–2^ K^–1^ per bidentate ligand (with the uncertainty indicating a 95% confidence interval). A bidentate ligand, thus, increases thermal conductance across the nanocrystal/ligand interface by 129 ± 63% on a per-ligand basis, which suggests that the number of contacts instead of number of ligands determines the magnitude of thermal conductance of the interface.

An analysis of IPEP experiments on thermal transport in colloidal suspensions show that the ligand/solvent interface is the bottleneck to heat transfer with organic solvents, having a thermal conductance that is 1 order of magnitude smaller than the conductance at the nanocrystal/ligand interface. A molecular dynamics model, validated by agreement with the experimental results, finds that bidentate bonds have higher conductance on a per-ligand basis, while the monodentate bonds are more plentiful as grafting densities increase. Our robust radial symmetric model could be used to analyze the heat transfer process in similar organic–inorganic systems where excitation occurs in the ligands. Isolating the interfacial thermal conductances provides insight that enhanced heat transfer will require engineering of the ligand/solvent interface.

## Supporting Information Available

Details of synthesis and IPEP experiments and details of ANSYS model/numerical methods and radial symmetric model/molecular dynamics simulation (PDF)
