# Supplementary material for: Differentiating Thermal Conductances at Semiconductor Nanocrystal/Ligand and Ligand/Solvent Interfaces in Colloidal Suspensions
Source: Nano Lett. 2023 Apr 24;23(9):3687–93. doi: 10.1021/acs.nanolett.2c04627 (PMC10176576; doi:10.1021/acs.nanolett.2c04627)
Supplement: Supplementary file 1 — nl2c04627_si_001.pdf [file nl2c04627_si_001.pdf]

Supplementary Information

Differentiating thermal conductances at semiconductor nanocrystal/ligand and ligand/solvent interfaces in colloidal suspensions

Yuxing Liang[1], Benjamin T. Diroll[2], Kae-Lin Wong[3], Samantha M. Harvey[2], Wee-Liat Ong[3*], Richard D. Schaller[2], Jonathan A. Malen[1*]

[1] *Department of Mechanical Engineering, Carnegie Mellon University, 5000 Forbes Ave, Pittsburgh, PA 15213, United States*

[2] *Center for Nanoscale Materials, Argonne National Laboratory, 9700 S Cass Ave, Lemont, IL 60439, United States*

[3] *ZJU-UIUC Institute, College of Energy Engineering, Zhejiang University, 718 East Haizhou Road, Hangzhou, 310058, China.*

*jonmalen@andrew.cmu.edu

*weeong@intl.zju.edu.cn

1. Details of synthesis and IPEP experiments

1.1 Materials. Cadmium acetylacetonate (99.9 %), selenium powder (99.99 %, 100 mesh), oleic acid (90 %), oleylamine (70 %), and octadecene (90 %) were purchased from Sigma-Aldrich. All solvents were ACS grade or higher.

1.2 Synthesis. Synthesis of all samples was performed using a modified version of ramped heating synthesis of zinc blende CdSe[1] in which all samples were exposed to the same chemicals. All reactions used 372 mg (2 mmol) of cadmium acetylacetonate as the cadmium precursor and 48 mg of selenium powder as the selenium precursor. For synthesis of 2.4 nm, 2.8 nm, 3.7 nm, and 4.7 nm samples, 800 µL, 800 µL, 1200 µL, and 1600 µL of oleic acid, respectively. After holding the reaction pot consisting of oleic acid, cadmium acetylacetonate, and 30 mL octadecene under vacuum at 120 °C for 1 hour, the reaction pot was cooled under nitrogen flow to ~50 °C, and the selenium powder was added under nitrogen counterflow. The reaction vessel was then heated to a desired set-point temperature, which was 215 °C, 240 °C, 240 °C, and 260 °C for 2.4 nm, 2.8 nm, 3.7 nm, and 4.7 nm samples, respectively. Upon reaching the set-point temperature, 2 mL of oleic acid and 2 mL of oleylamine were injected and the reactions proceeded for 5 minutes (2.4 nm) or 30 minutes (others). Reactions were cooled to ~50 °C by removing the heating mantle and diluted with 30 mL of toluene at that temperature.

Purification of reaction batches was performed by separately decanting the crude reaction contents into four different centrifuge tubes to which antisolvent was added. Each centrifuge tube was treated with enough acetone, methanol, isopropanol, or ethanol to induce flocculation, then centrifuged. After decanting the colorless supernatant, the colored pellet was redispersed in clean toluene. This cycle of precipitation and dispersal was repeated six times with aliquots for measurement taken after two, four, and six cycles. Aliquots in toluene were evaporated under nitrogen flow, dispersed in carbon tetrachloride, evaporated again,

then dispersed again in carbon tetrachloride. Samples for optical measurements were diluted from this stock and samples for thermal analysis were dried from this stock.

Figure S1 shows the infrared absorption of pure oleylamine and oleic acid as well as the several 2.8 nm CdSe nanocrystal samples. The data indicates that oleic acid, which has a strong carbonyl stretch which shifts from the free acid at 1720 cm^{-1} to the bound stretch at 1535 cm^{-1}. In the isolated particles there is not a unique vibration associated with oleylamine (at 1620 cm^{-1}) nor substantial carbonyl stretch from free oleic acid. This supports an oleate dominant ligand shell.

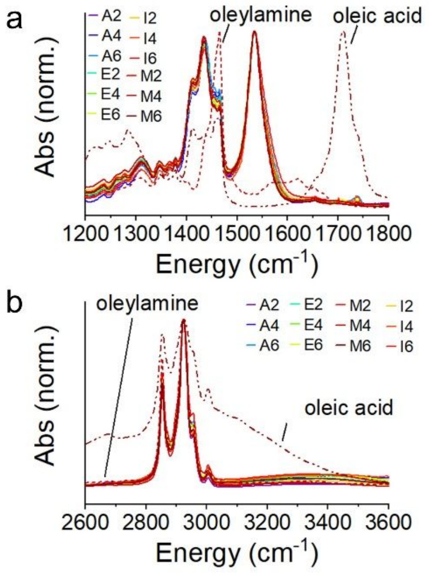

Figure S1. (a) Fingerprint region of infrared absorption of solid films of 2.8 nm CdSe nanocrystals under different washing conditions: A indicates acetone antisolvent, E ethanol, M methanol, and I isopropanol; the number indicates the number of antisolvent precipitations with the chosen antisolvent. Pure oleic acid and oleylamine spectra are also shown in dashed lines. These were collected by sandwiching pure liquid between pressed CaF_2 plates. (b) The same samples measured at the C-H stretching region.

1.3 Spectroscopy. Static, temperature-dependent absorption measurements were performed using samples loaded into a 1 cm cuvette in a cuvette heating adaptor for a Cary-60 spectrometer. Samples were permitted to equilibrate for 5 minutes at each temperature jump before measurement. Data was collected from three averaged scans with 0.5 nm intervals and an integration time of 0.5 s per point.

Fourier-transform infrared spectra were collected for selected samples dissolved in carbon tetrachloride within a liquid cell with calcium fluoride windows. Data was collected using a Thermo-Fisher Nicolet 6700 FT-IR.

Transient infrared pump, electronic probe (IPEP) measurements were performed by splitting the output of a 35 fs amplified Ti: sapphire laser (SpectraPhysics) to generate pump and probe beams. The pump excitation was generated at 3.5 μm using an optical parametric amplifier; the probe beam was generated by focusing the 800 nm fundamental output into a sapphire crystal to generate a white light supercontinuum. Temporal delay was achieved by using an optical delay stage on the probe beam. To generate transient spectra, the pump beam was chopped at 1 kHz and pump and probe beams were spatiotemporally overlapped at the sample.

Dynamics of the temperature evolution for each of the samples under different solvent washing conditions (*e.g.* A2 for washing with acetone two cycles) are shown in Figures S1.)

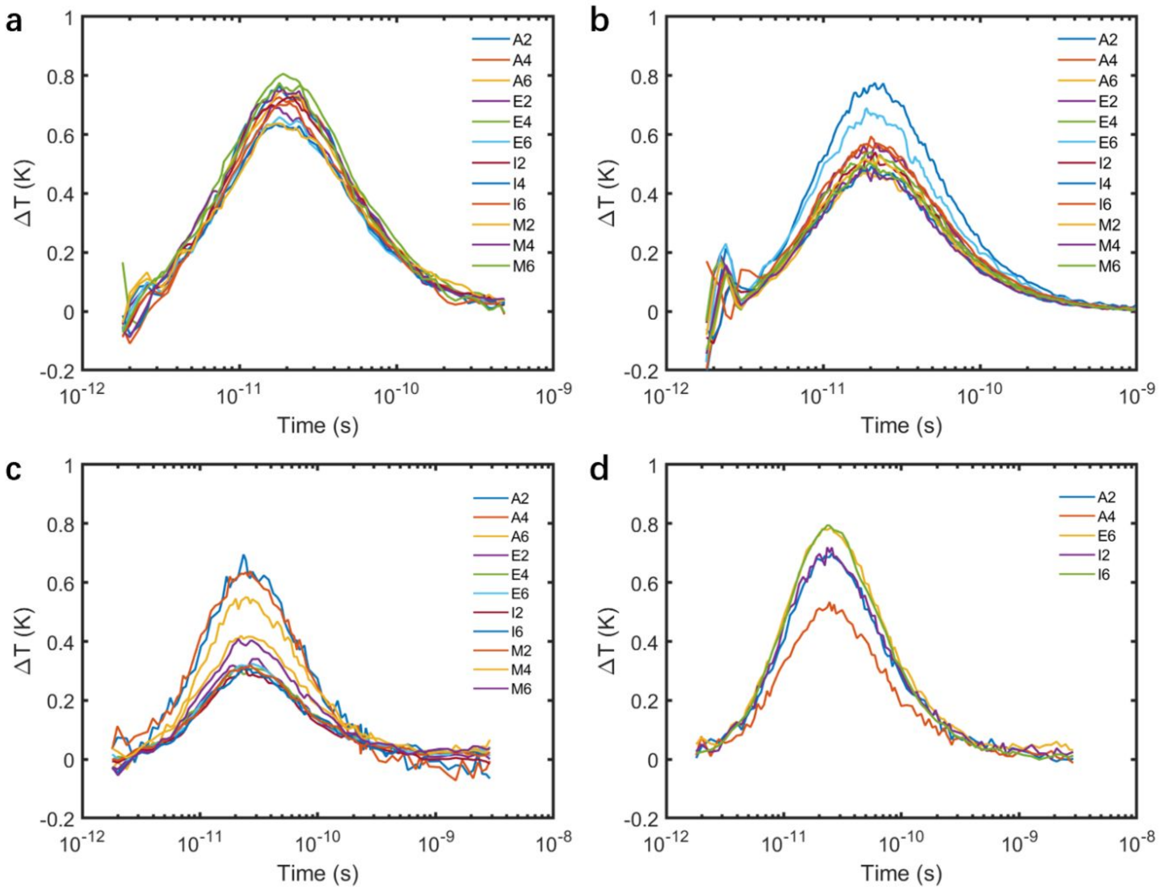

Figure S2. Dynamics of IPEP measurements for (a) 2.4 nm, (b) 2.8 nm, (c) 3.7 nm, and (d) 4.7 nm CdSe nanoparticles. Legend labels indicate the antisolvent used in precipitation purifications: A = acetone, E = ethanol, I = isopropanol, M = methanol. Legend label numbers indicate the number of precipitation and redispersion cycles.

The ligand cross section was estimated by first collecting the gravimetric analysis of the weight loss from heating to estimate the graft density of oleate ligands on the surface of the CdSe nanocrystals with defined quasi-spherical size. Several 2.8 nm CdSe samples were measured by visible absorption spectroscopy in a 1 mm CaF_2 cell and then measured in a FT-IR to compare the intensity of absorption for the CdSe nanocrystals. The molar extinction (7.2×10^5 $M^{-1}cm^{-1}$) of the samples was obtained from literature[2]. This was converted to a cross section of 2.8×10^{-16} using the sizing estimate of the sample from TEM imaging according to

$$\sigma = \frac{1000 \cdot \ln(10) \cdot \varepsilon}{N_A}$$

in which N_A is Avogadro's number. The absorbance of the nanocrystals can then be compared to the to the collective absorption of the oleate ligands (here between 60 and 82 ligands per nanocrystal) apparent in the FT-IR absorption for several samples. The result of this comparison for one sample is shown in Figure S3. The cross-section spectrum of the C-H stretches reported above was estimated by dividing the collective cross section of the oleate ligands by their number and averaging the result from several samples, with the estimated peak cross section is per ligand of ~$1.8\pm0.5\times10^{-18}$ cm^2 at 3420 nm.

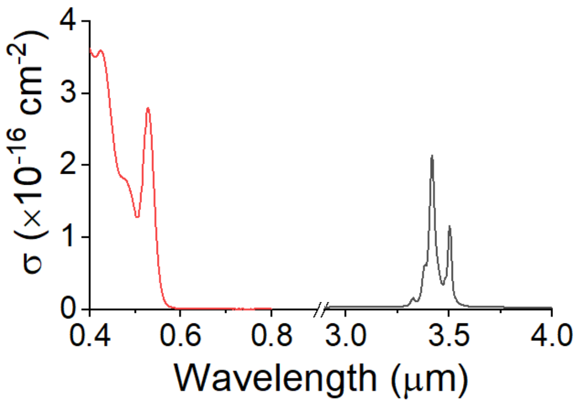

Figure S3. Absorption in visible and infrared of the same 2.8 nm CdSe nanocrystal sample with an average graft density of 3.3 nm^{-2} and a total number of ligands of 82 per particle.

A simple estimate for the upper bound number of absorption events, the mean measured intensity of the pulsed pump beam (1 kHz) was 5.78 mW, or 5.78 μJ/pulse with photons centered at 3450 nm (5.7×10^{-20} J) and a spot size of ~420 μm diameter (0.00138 cm^2, defined as the decay of the gaussian beam to 1/e of peak intensity), resulting in a photon density of ~7.2×10^{16} cm^{-2} per pulse. The probability of absorption by any given ligand is the cross section multiplied by the photon density per pulse, here ~0.13 absorption events per ligand per pulse. This overestimates the number of absorption events because the peak absorption intensity of the oleate ligand exist only over a narrow range of wavelength and the pump beam is not monochromatic as shown in Figure S4.

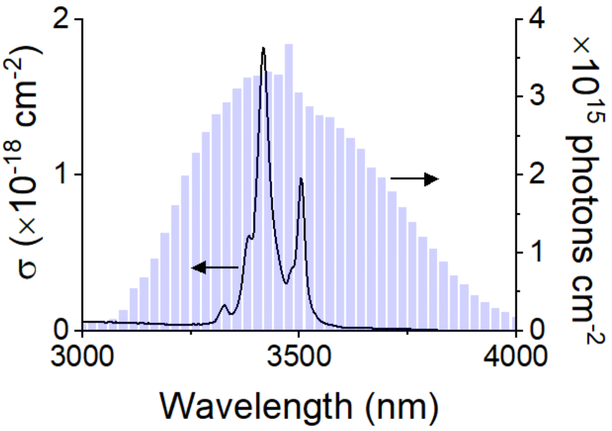

Figure S4. Comparison of estimated cross section of oleate, left axis in black, versus the measured pump spectrum of infrared pump, right axis in blue, with estimated photons cm^{-2} for each pixel (24 nm) with a total photon flux of ~7.2 ×1016 cm^{-2}, which is the value estimated based upon the average photon energy. The total photon flux distribution was estimate according to the spectrum of detected counts using a mercury cadmium telluride array, assuming that counts at any given pixel represented the same number of incident photons.

The estimated number of photons per pulse is integrated over the pulse bandwidth, which includes many photons for which the oleate ligands have very low cross section. Taking the distribution of the photon flux as a function of photon wavelength, as measured using a mercury cadmium telluride CCD and shown in Figure S4, and multiplying this with the estimated oleate cross section (Figure S5), yields a collective probability of ~0.019 absorption events per ligand. This value is somewhat lower than the trendline for the local minimum solution to the data presented in the main text.

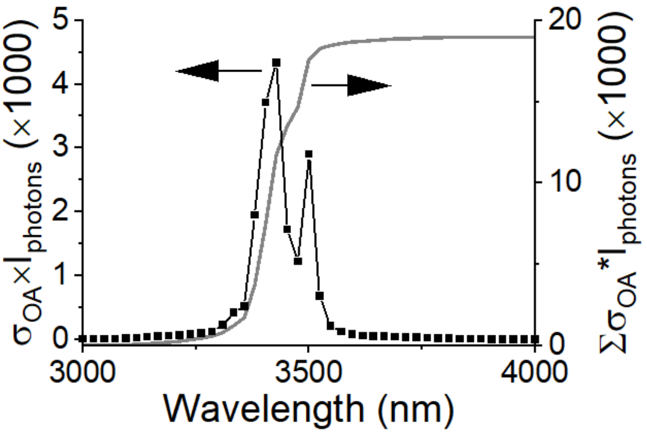

Figure S5. Wavelength-dependent probability of absorption of the IR pump beam by an oleate ligand. The sum of all probabilities is 0.019 as shown on the right axis.

1.4 Structural characterization. Transmission electron microscopy imaging was performed using a JEOL 2100F TEM. Sizing analysis was performed using ImageJ software. Sizing estimates for the samples were generated from statistical analysis of TEM micrograph data. X-ray diffraction was collected using a Bruker D2 phaser tool.

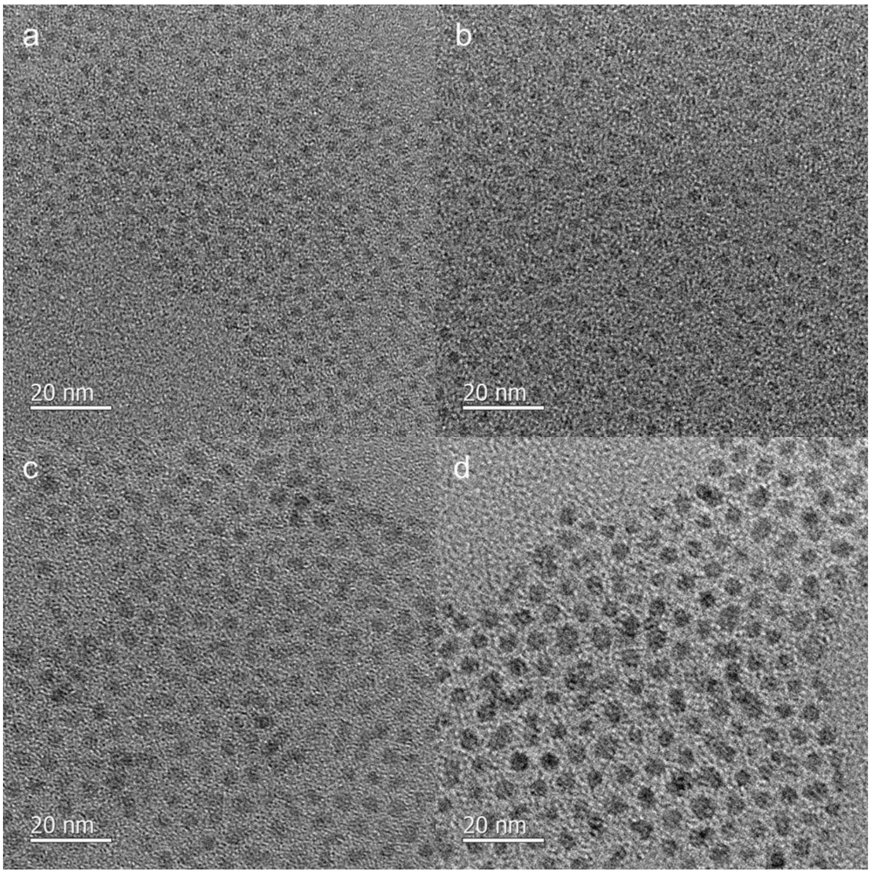

Figure S6. Transmission electron microscope (TEM) images of 2.4, 2.8, 3.7, and 4.7 nm average diameter CdSe nanocrystals.

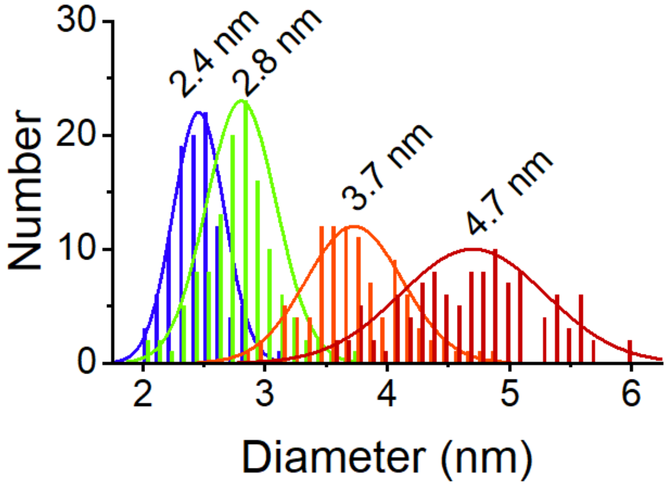

Figure S7. Sizing histograms of CdSe nanocrystal samples from measurement of TEM images.

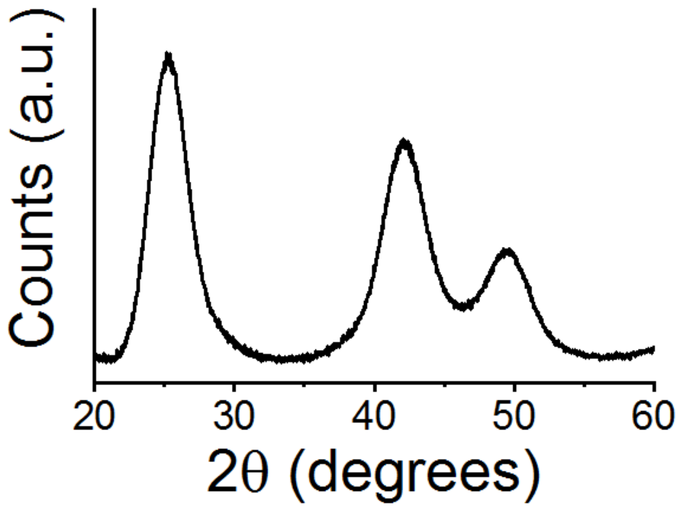

Figure S8. Powder X-ray diffraction pattern of 2.8 nm CdSe nanocrystals.

1.5 Thermal analysis. Thermogravimetric analysis was performed on samples (typically 2-4 mg) dried into aluminum pans using a Netzsch Jupiter F3 STA. Samples were heated from room temperature and equilibrated at 50 °C, then heated under air to 500 °C. Mass loss during the heating process was ascribed to combustion of ligands.

2. Details of ANSYS model

2.1 Geometry. A sphere and two concentric shells are developed with diameters of 4.7nm, 9.7nm and 110nm in 'DesignModeler'. Since the nanocrystals are assumed to be uniformly distributed in the solvent, the outer radius of the solvent domain R_{out} is calculated based on their number density η during the measurement, as expressed by $\frac{4}{3}\pi R_{out}^{3} = \frac{1}{\eta}$. In order to generate a radial symmetric mesh which is numerically stable, the sphere is cut into 8 equal parts in the 'Geometry'. We are still able to apply heat generation to specific elements of one octant, despite generating the mesh in this way. The materials properties are listed in Table S1.

2.2 Model. 'Transient Thermal Module' is used to develop the model. The thermal conductance is defined in 'Connections-Contacts-Thermal conductance'. The Multizone Method is applied to all objects. Inflation is applied to the solvent layer with the contact surface between solvent and ligands. The Tetrahedrons Method is applied to the nanocrystal layer. The initial temperature is set at 22℃. 27 elements of mesh in the intermediate shell are selected to input the heat ($\dot{q}$) with 'Internal Heat Generation'. 400 time steps are defined in the time domain of 0-1000ps. The transient solver used implicit time steps. The simulation results are converged with larger number of timesteps or finer mesh and it is confirmed that with the current settings, the equations are numerically stable.

2.3 Parametric study. The APDL command is used to conduct the parametric study. 221 independent simulations with 221 combinations of h_{NC-lig} and $h_{lig-sol}$ in the range of 0-200 $MW\cdot m^{-2}\cdot K^{-1}$ are run automatically with the ANSYS datafile. Output includes the temperature of 2367 nodes of the nanocrystal at 400 timesteps. The average temperature of the nanocrystal can be obtained by the post processing process.

Table S1. Parameters

	Nanocrystals[3]	Ligands[4,5]	Solvent[6]
$\boldsymbol{Radius/length}$	$2.35\ nm$	$2.5\ nm$	$55\ nm$
$\boldsymbol{Density}$	$5820\ Kg/m^3$	$232.4\ Kg/m^3$	$1620\ Kg/m^3$
$\boldsymbol{Heat\ capacity}$	$271\ J/(Kg * K)$	$1956\ J/(Kg * K)$	$887.4\ J/(Kg * K)$
$\boldsymbol{Thermal\ conductivity}$	$4\ W/(K * m)$	$0.224\ W/(K * m)$	$0.111\ W/(K * m)$

3. Numerical methods of the radial symmetric model

3.1 Analytical solution to the two-temperature model

The original differential equations of the heat transfer can be expressed as:

$$h_{NC-lig}A_{NC-lig}(\theta_{NC}-\theta_{lg})+h_{lig-sol}A_{lig-sol}(\theta_{sol}-\theta_{lig})+\dot{q}=m_{lig}c_{lig}\frac{d\theta_{lig}}{dt},\#(1)$$

$$h_{NC-lig}A_{NC-lig}(\theta_{lig}-\theta_{NC})=m_{NC}c_{NC}\frac{d\theta_{NC}}{dt},\#(2)$$

using θ we substitute for the temperature differences,

$$\theta_{lig}=T_{sol}-T_{lig},\#(3)$$

$$\theta_{NC}=T_{sol}-T_{NC}.\#(4)$$

Then we can further reduce the equations to the matrix form:

$$\dot{\boldsymbol{\theta}}=\begin{bmatrix}-\frac{h_{NC-lig}A_{NC-lig}}{m_{lig}c_{lig}}-\frac{h_{lig-sol}A_{lig-sol}}{m_{lig}c_{lig}} & \frac{h_{NC-lig}A_{NC-lig}}{m_{lig}c_{lig}}\\ \frac{h_{NC-lig}A_{NC-lig}}{m_{NC}c_{NC}} & -\frac{h_{NC-lig}A_{NC-lig}}{m_{NC}c_{NC}}\end{bmatrix}\boldsymbol{\theta}+\begin{bmatrix}-\frac{\dot{q}}{m_{lig}c_{lig}}\\ 0\end{bmatrix}.\#(\mathbf{5})$$

It is clear shown that the system can be treated to the homogeneous part and nonhomogeneous part: $\dot{\boldsymbol{\theta}}=\boldsymbol{B\theta}+\boldsymbol{g}(t)$**.** We will first look into the general solution of the homogeneous system $\dot{\boldsymbol{\theta}}=\boldsymbol{B\theta}$. We find that the eigenvalues of $\boldsymbol{B}$, r_1 and r_2 and that the corresponding eigenvectors $\begin{bmatrix}b_{11}\\ b_{21}\end{bmatrix}$ and $\begin{bmatrix}b_{12}\\ b_{22}\end{bmatrix}$. Thus, the general solution of the homogeneous system is:

$$\boldsymbol{\theta}_h=c_1\begin{bmatrix}b_{12}\\ b_{22}\end{bmatrix}e^{r_1t}+c_2\begin{bmatrix}b_{12}\\ b_{22}\end{bmatrix}e^{r_2t}=\boldsymbol{\Psi}(t)\boldsymbol{c}.\#(6)$$

where $\boldsymbol{\Psi}(t)$ is a fundamental matrix

$$\boldsymbol{\Psi}(t)=\begin{bmatrix}b_{11}e^{r_1t} & b_{21}e^{r_2t}\\ b_{21}e^{r_1t} & b_{22}e^{r_2t}\end{bmatrix}.\#(7)$$

The particular solution of nonhomogeneous system can be expressed as $\boldsymbol{\theta}=\boldsymbol{\Psi}(t)\boldsymbol{u}(t)$, where $\boldsymbol{u}(t)$ is the unknown vector. If we plug the particular solution back to the original equation (Equation 5.), we obtain,

$$\boldsymbol{\Psi}(t)\boldsymbol{u}'(t)=\boldsymbol{g}(t),$$

$$\begin{bmatrix}b_{11}e^{r_1t} & b_{21}e^{r_2t}\\ b_{21}e^{r_1t} & b_{22}e^{r_2t}\end{bmatrix}\begin{bmatrix}u_1'\\ u_2'\end{bmatrix}=\begin{bmatrix}-\frac{\dot{q}}{m_{lig}c_{lig}}\\ 0\end{bmatrix}=\begin{bmatrix}-ke^{-\frac{t}{\tau}}\\ 0\end{bmatrix}.\#(8)$$

Solving by row reduction, we obtain

$$\begin{cases} u_1' = -\dfrac{b_{22}k}{b_{11}b_{22}-b_{12}b_{21}} e^{-\left(\frac{1}{\tau}+r_1\right)t} \\ u_2' = \dfrac{b_{21}k}{b_{11}b_{22}-b_{12}b_{21}} e^{-\left(\frac{1}{\tau}+r_2\right)t} \end{cases} \xrightarrow{integrate} \begin{cases} u_1(t) = \dfrac{b_{22}k}{\left(\frac{1}{\tau}+r_1\right)(b_{11}b_{22}-b_{12}b_{21})} e^{-\left(\frac{1}{\tau}+r_1\right)t} \\ u_2(t) = -\dfrac{b_{21}k}{\left(\frac{1}{\tau}+r_2\right)(b_{11}b_{22}-b_{12}b_{21})} e^{-\left(\frac{1}{\tau}+r_2\right)t} \end{cases} .(9)$$

The components of the general solution of the nonhomogeneous linear system are summed,

$$\boldsymbol{\theta} = c_1\begin{bmatrix} b_{11} \\ b_{21} \end{bmatrix} e^{r_1 t} + c_2\begin{bmatrix} b_{12} \\ b_{22} \end{bmatrix} e^{r_2 t} + \begin{bmatrix} b_{11}e^{r_1 t} & b_{12}e^{r_2 t} \\ b_{21}e^{r_1 t} & b_{22}e^{r_2 t} \end{bmatrix} \begin{bmatrix} \dfrac{b_{22}k}{\left(\frac{1}{\tau}+r_1\right)(b_{11}b_{22}-b_{12}b_{21})} e^{-\left(\frac{1}{\tau}+r_1\right)t} \\ -\dfrac{b_{21}k}{\left(\frac{1}{\tau}+r_2\right)(b_{11}b_{22}-b_{12}b_{21})} e^{-\left(\frac{1}{\tau}+r_2\right)t} \end{bmatrix} .(10)$$

The unknowns parameter c_1 and c_2 can be determined with the initial conditions. Here, the initial condition is $\boldsymbol{\theta_0} = \begin{bmatrix} 0 \\ 0 \end{bmatrix}$, which indicates that at $t = 0s$, the temperature difference between nanoparticle and the environment and that between ligand and the environment are zero.

When $t = 0s$, Equation 9 can be written as:

$$\boldsymbol{\theta_0} = c_1\begin{bmatrix} b_{11} \\ b_{21} \end{bmatrix} + c_2\begin{bmatrix} b_{12} \\ b_{22} \end{bmatrix} + \begin{bmatrix} b_{11} & b_{21} \\ b_{21} & b_{22} \end{bmatrix} \begin{bmatrix} \dfrac{b_{22}k}{\left(\frac{1}{\tau}+r_1\right)(b_{11}b_{22}-b_{12}b_{21})} \\ -\dfrac{a_{21}k}{\left(\frac{1}{\tau}+r_2\right)(b_{11}b_{22}-b_{12}b_{21})} \end{bmatrix} = \begin{bmatrix} 0 \\ 0 \end{bmatrix},\#(11)$$

$$\boldsymbol{c} = \begin{bmatrix} b_{11} & b_{21} \\ b_{21} & b_{22} \end{bmatrix} \backslash \left(\begin{bmatrix} b_{11} & b_{21} \\ b_{21} & b_{22} \end{bmatrix} \begin{bmatrix} \dfrac{b_{22}k}{\left(\dfrac{1}{\tau} + r_1\right)(b_{11}b_{22} - b_{12}b_{21})} \\ -\dfrac{b_{21}k}{\left(\dfrac{1}{\tau} + r_2\right)(b_{11}b_{22} - b_{12}b_{21})} \end{bmatrix} \right). \#(12)$$

3.2 Numerical approach to solve the radial symmetric model

With the addition of the solvent, the ordinary differential equations become a partial differential equation-ordinary differential equation system,

$$\frac{1}{r^2}\frac{\partial}{\partial r}\left(r^2 \cdot k_{sol} \cdot \frac{\partial \theta_{sol}}{\partial r}\right) = \rho_{sol} c_{sol} \frac{\partial \theta_{sol}}{\partial t},\ R \le r \le R_{out}.\#(13)$$

In order to solve this equation, the spatial domain of the solvent is discretized into n shells with central difference method as shown in Figure 2b.1. The thickness of each shell is $\Delta r = \frac{r_{sol}}{n+1}$ and central difference results in the following approximations of the derivatives,

$$\frac{\partial \theta_{sol}}{\partial r} = \frac{\theta_{sol}^{i+1}(t) - \theta_{sol}^{i-1}(t)}{2 \cdot \Delta r},\ i = 1, 2, 3 \ldots n,\#(14)$$

$$\frac{\partial^2 \theta_{sol}}{\partial r^2} = \frac{\theta_{sol}^{i+1}(t) - 2 \cdot \theta_{sol}^{i}(t) + \theta_{sol}^{i-1}(t)}{(\Delta r)^2},\ i = 1, 2, 3 \ldots n.\#(15)$$

Therefore the partial differential equation can be written as a series of ordinary differential equations as follows,

$$\left(1 - \frac{\Delta r}{r_i}\right)\theta_{sol}^{i+1} - 2 \cdot \theta_{sol}^{i} + \left(1 + \frac{\Delta r}{r_i}\right)\theta_{sol}^{i-1} = \frac{\rho_{sol} c_{sol} (\Delta r)^2}{k_{sol}} \frac{d\theta_{sol}^{i}}{dt},\ i = 1, 2, 3 \ldots n.\#(16)$$

The boundary condition at the ligand/solvent interface is then written as:

$$h_{lig-sol}\left(\theta_{sol}^{1} - \theta_{lig}\right) = k_{sol}\frac{\theta_{sol}^{2} - \theta_{sol}^{1}}{\Delta r}.\#(17)$$

At the edge side of the spatial domain, the heat flux is zero such that the temperature difference does not change,

$$\theta_{sol}^{n} = \theta_{sol}^{n+1}.\#(18)$$

For n=50, the ordinary differential system is then solved by MATLAB with both the analytical solution (same as section 3.1) and also the numerical built-in solver ode45. The ode45 solver is numerically stable compared to the analytical method with faster speed.

3.3 Uncertainty of the fitted thermal conductance from the radial symmetric model

The uncertainty of the fitted thermal conductance is estimated based on a repetitive fitting algorithm.

1. Find the best fit by minimizing $\chi^2 = (T_i - T(t_i))^2$ over all timesteps
2. Adjust the parameter β, whose uncertainty we wish to determine, to a new value of β'.
3. Re-minimize χ^2 by adjusting the other parameters
4. Iterate Step 2 and 3 until the value of the re-minimized χ^2 is 20% larger than the χ^2 in Step 1.
5. Report the uncertainty in that parameter as $|\beta - \beta'|$.

3.4 Discussion on the best fit to the experiment data

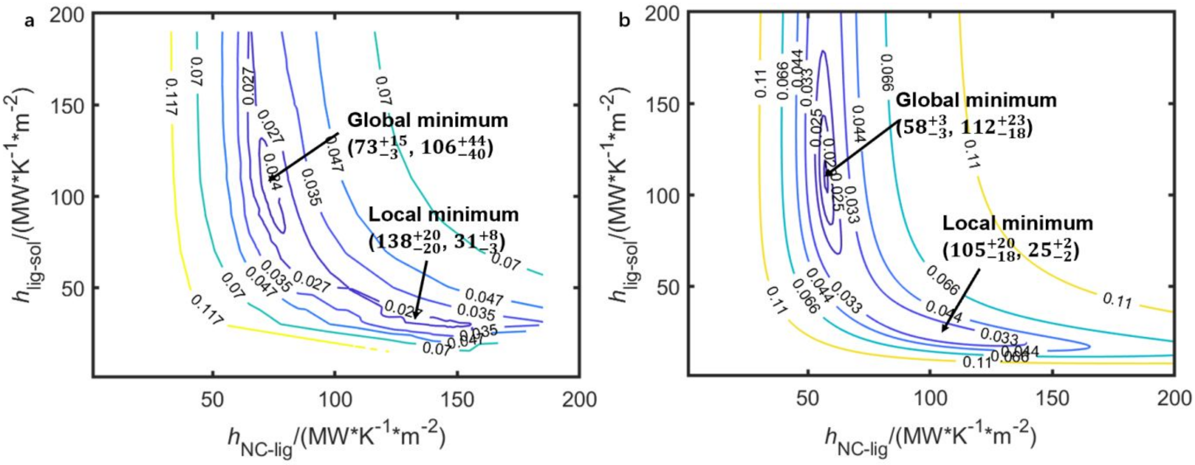

Figure S9**.** Root mean squared error contour model with thermal conductances in the range of 0-200 MW·m^{-2}·K^{-1} from (a) ANSYS model; (b) radial symmetric model. Global and local minima are labeled.

We first fit the data to the normalized temperature change to reduce the degrees of freedom, and thus minimize our uncertainty. When fitting to the normalized data, the only undefined parameters in the heat transfer model are the two targeted thermal conductances. When fitting

to the non-normalized temperature profile, the average number of photons absorbed needs to be fitted as well, which adds an additional degree of freedom that can result in large uncertainty for all three parameters. Hence, we first fit the thermal conductances to the normalized data, then use those values in a non-normalized model to match the non-normalized temperature data by varying the number of photons absorbed.

When fitting to the normalized data, the contours of the root mean squared error based on the radial symmetric model reveal two minima (shown in Figure S9b). The global minimum is $h_{NC-lig} = 58^{+3}_{-3}$ MW·m^{-2}·K^{-1}, $h_{lig-sol} = 112^{+23}_{-18}$ MW·m^{-2}·K^{-1} while the local minimum is $h_{NC-lig} = 105^{+20}_{-18}$ MW·m^{-2}·K^{-1}, $h_{lig-sol} = 25^{+2}_{-2}$ MW·m^{-2}·K^{-1}.

Like radial symmetric model, the ANSYS model finds two fitting minima that imply opposite conclusions (shown in Figure S9a) i.e. that either $h_{NC-lig} > h_{lig-sol}$ or $h_{NC-lig} < h_{lig-sol}$. The global minimum, $\chi = 0.0216$, corresponds to $h_{NC-lig} = 73$ MW·m^{-2}·K^{-1}, $h_{lig-sol} = 106$ MW·m^{-2}·K^{-1}. A second local minimum with χ approximately 32% larger than the global minimum corresponds to $h_{NC-lig} = 138$ MW·m^{-2}·K^{-1}, $h_{lig-sol} = 31$ MW·m^{-2}·K^{-1}. The contours from this model are similar to those from radial symmetric model and the minima are within 20% of those predicted by radial symmetric model for this dataset.

The magnitude of energy deposited in the organic ligands is examined to help us learn more about the heat transfer process under these two opposite conclusions. While normalized temperature rise was used for fitting to determine the conductances, we use the absolute temperature rise to determine the number of photons which must be absorbed under the different interfacial conductance minima identified by the models. The photon energy of the pump laser is known from the spectrum (Figure S3). Then the magnitude of the heat generation rate is given by Equation 19 based on conservation of energy.

$$nhv = \int \dot{q}dt,\#(19)$$

Where n is the number of absorbed photons, h is Planck's constant, v is the wavelength of the pump laser and $\dot{q}$ is the heat generation rate. Then the average number of absorbed photons can be predicted from Equation 19 by fitting $\dot{q}$ to the non-normalized experimental data with the known thermal conductances.

The number of photons absorbed in the experiments are plotted in Figure S10 versus number of ligands bound to the nanocrystals. Both the plots with the results from the global minimum and the local minimum show a positive correlation intuitively indicating that more photons will be absorbed if there are more ligands. The linear fit to the global minimum points has a large positive intercept, which is non-physical because we expect the ligands to have a constant cross section and thus no excitation should exist when there are no ligands. The linear fit to the local minimum points has an intercept of zero, within our uncertainty and suggest an acceptable solution. The estimated cross section from the experiment also agrees with the local minimum result as detailed in the Section 1.3. Therefore, we conclude that the conductances associated with the *local minimum* are correct, i.e. $h_{NC-lig} > h_{lig-sol}$.

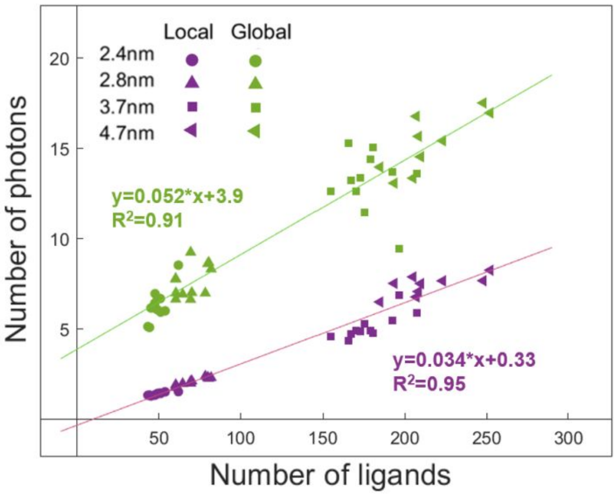

Figure S10**.** The plot of the number of photons absorbed versus the number of ligands on the NC based on the thermal conductances given by the global minimum and local minimum respectively (see Figure S9).

4 Molecular dynamics simulation

4.1 Calculation of specific heat capacity (c_p)

Molecular dynamics simulations were performed using the Open-source Large-scale Atomic-molecular Massively Parallel Simulator (LAMMPS) package [7]. Fifty oleic acid molecules in a 2x2x2 nm^3 box were used to calculate the heat capacity of the ligands under the OPLS-AA potential. The structure was relaxed through a series of steps by heating the system to 310 K in an NVT ensemble, followed by an NPT ensemble to stabilize the pressure to 0 atm, before equilibrating in an NVE ensemble for five nanoseconds each. Next, the system enthalpy was calculated at 290 K ($T - \Delta T$) and 330 K ($T + \Delta T$) under a constant pressure of 0 atm in an NPT ensemble for ten nanoseconds. The specific heat capacity value was estimated using the derivatives of the enthalpy [8,9]:

$$c_p = \frac{H(p,T + \Delta T) - H(p,T - \Delta T)}{2\Delta T}, \#(20)$$

where c_p, $H, p, T, and\ \Delta T$ refers to the specific heat capacity, enthalpy, pressure, temperature, and temperature difference. The MD simulation results were averaged over eight runs (Figure S11) to minimise the statistical fluctuations.

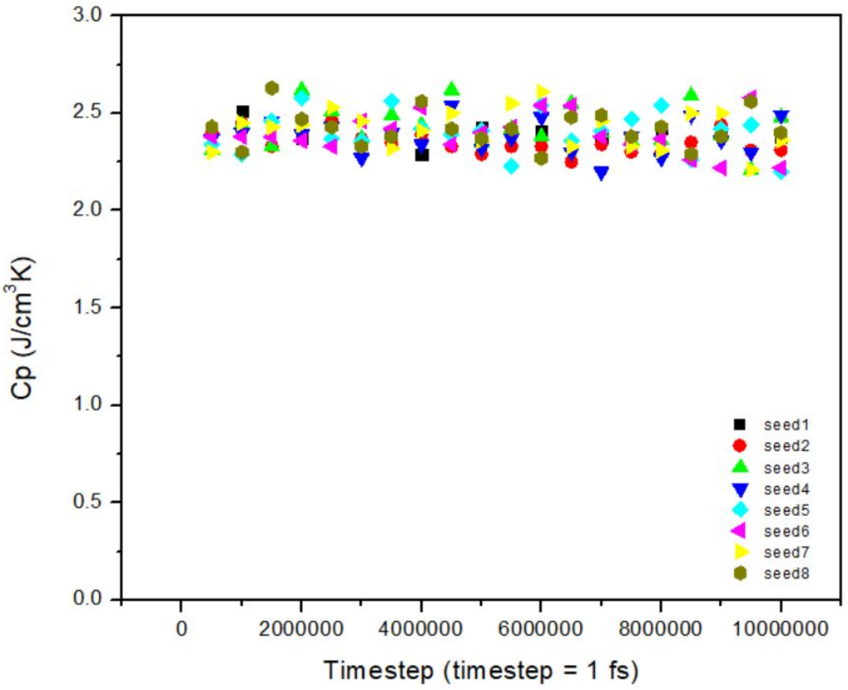

Figure S11. The simulated specific heat capacity values at different timesteps for eight different runs.

The averaged specific heat capacity of oleic acid molecules at 310 K is calculated to be 2.39 J/cm^3K. This average c_p value agrees with other calculated values in the literature [10,11].

4.2 Calculation of the interfacial thermal conductance (*G*)

Our model consists of a sphere-like CdSe core of 1.6 nm in radius (324 Cd and 318 Se atoms) with oleic acid molecules attached as ligands. The CCl_4 molecules act as the solvent around this nanocrystal. A stiff spring (i.e., k =100 $kcal/(molA^2)$) at the core center restrained the nanocrystal to the center of the simulation box. A different number of oleic acid ligands were randomly positioned vertically on the nanocrystal surface to investigate the effect of grafting densities on thermal conductance. At least 3000 CCl_4 molecules were randomly distributed around the nanocrystal in an 8 nm x 8 nm x 8 nm simulation box. Periodic boundary

conditions were applied in all three spatial directions. Figure 4c shows a cross-sectional view of the CdSe core with 100 oleic acid ligands surrounded by CCl_4 molecules.

The CdSe nanocrystal was modeled using the Lennard-Jones (LJ) potential and long-range Coulombic interactions with parameters shown in Table S2 [12]. The potential for the interactions between Cd and Se atoms is shown in Equation 21.

$$U_{ij}(r_{ij}) = \sum_{i}^{a}\sum_{j}^{b}\frac{1}{4\pi\varepsilon_0}\frac{q_i q_j}{r_{ij}} + 4\varepsilon_{ij}\left[\left(\frac{\sigma_{ij}}{r_{ij}}\right)^{12} - \left(\frac{\sigma_{ij}}{r_{ij}}\right)^{6}\right], \#(21)$$

where ε_0 refers to the dielectric constant of vacuum, r_{ij} is the distance between atoms *i* and j, q_i and q_j are the electric charge of atom *i* and atom j respectively.

Table S2. The LJ parameters for non-bonding interactions in the CdSe nanocrystal

Interaction type	ε_{ij} $(kcal/mole)$	σ_{ij} (Å)
Cd – Cd	0.07400	1.234
Cd - Se	0.36389	2.940
Se - Se	0.10196	4.852

The OPLS-AA potentials were used to describe the interactions in and between oleic acid ligands and CCl_4 solvents [13], which included bond, angle, dihedral, and improper potentials [14–17]:

$$U_{ij}(r_{ij}) = \sum_{i}^{a}\sum_{j}^{b}\frac{1}{4\pi\varepsilon_0}\frac{q_i q_j}{r_{ij}} + 4\varepsilon_{ij}\left[\left(\frac{\sigma_{ij}}{r_{ij}}\right)^{12} - \left(\frac{\sigma_{ij}}{r_{ij}}\right)^{6}\right]$$

$$+ \sum_{bonds} K_b(b - b_0)^2$$

$$+ \sum_{angles} K_\theta(\theta - \theta_0)^2$$

$$+\sum_{dihedrals}\frac{1}{2}K_1[1+\cos(\phi)]+\frac{1}{2}K_2[1+\cos(2\phi)]+\frac{1}{2}K_3[1+\cos(3\phi)]+\frac{1}{2}K_4[1+\cos(4\phi)]$$

$$+\sum_{impropers}K_\phi[1+\mathrm{d}\cos(n\phi)] \quad (22)$$

The variables b, θ, and ϕ represents the bond length, angle, and improper dihedral angle, respectively. The parameters used are summarized in Table S3.

Table S3. Intermolecular parameters and vibrational parameters for oleic acid ligand and CCl_4 solvent [13]

Intermolecular parameters		
Interaction type	ε $(kcal/mole)$	σ (Å)
C − C	0.100	3.410
Cl − Cl	0.285	3.450
C_1-C_1	0.066	3.500
C_2-C_2	0.076	3.550
C_3-C_3	0.070	3.550
O_1-O_1	0.210	2.960
O_2-O_2	0.170	3.120
H_1-H_1	0.030	2.500
H_2-H_2	0.000	0.000

Intramolecular parameters																
Molecule	Interaction type	K_b $(kcal/mole)$	b_0 (Å)	Interaction type	K_θ $(kcal/mole)$	θ(Å)	Interaction type	K_1 $(kcal/mole)$	K_2 $(kcal/mole)$	K_3 $(kcal/mole)$	K_4 $(kcal/mole)$	Interaction type	K_ϕ $(kcal/mole)$	d	n
CCl_4	$C-Cl$	630.00	1.766	$Cl-C-Cl$	49.00	109.50									
Oleic acid	C_1-C_1	268.00	1.529	$C_1-C_1-C_1$	58.35	112.70	$C_1-C_1-C_1-C_1$	1.300	-0.200	0.200	0.000	$C_3-C_1-O_1-O_2$	10.500	-1	2
	C_1-C_2	317.00	1.510	$C_1-C_1-C_2$	63.00	111.10	$C_2-C_2-C_1-C_1$	0.346	0.405	-0.904	0.000	$C_1-C_1-H_1-H_1$	0.000	-1	2
	C_2-C_2	549.00	1.340	$C_1-C_2-C_2$	70.00	124.00	$C_1-C_2-C_2-C_1$	0.000	14.000	0.000	0.000	$C_2-C_2-H_1-C_1$	2.500	-1	2
	C_1-C_3	317.00	1.522	$C_1-C_3-O_1$	80.00	120.40	$C_3-C_1-C_1-C_1$	-2.060	-0.313	0.315	0.000				
	C_3-O_1	570.00	1.229	$C_1-C_3-O_2$	70.00	108.00	$O_1-C_3-C_1-C_1$	0.000	0.000	0.000	0.000				
	C_3-O_2	450.00	1.364	$C_2-C_1-H_1$	37.50	110.70	$O_2-C_1-C_1-C_1$	0.000	0.000	0.300	0.000				
	C_1-H_1	340.00	1.090	$C_1-C_2-H_1$	35.00	117.00	$H_2-O_2-C_3-C_1$	1.500	5.500	0.000	0.000				
	C_2-H_1	340.00	1.080	$C_2-C_2-H_1$	35.00	120.00	$H_1-C_1-C_1-C_2$	0.000	0.000	0.366	0.000				
	O_2-H_2	553.00	0.945	$C_2-C_1-H_1$	35.00	109.50	$H_2-O_2-C_3-O_1$	0.000	5.500	0.000	0.000				
				$C_3-O_2-H_2$	35.00	113.00	$H_1-C_1-C_1-C_1$	0.000	0.000	-0.100	0.000				
				$H_1-C_1-H_1$	33.00	107.80	$H_1-C_2-C_1-C_1$	0.000	-8.000	0.000	0.000				
				$O_1-C_3-O_2$	80.00	121.00	$H_1-C_2-C_2-C_1$	0.000	14.000	0.000	0.000				
							$H_1-C_1-C_2-C_2$	0.000	0.000	-0.372	0.000				
							$O_2-C_3-C_1-C_1$	1.000	0.546	0.450	0.000				
							$H_1-C_1-C_2-H_1$	0.000	0.000	0.318	0.000				

Molecular dynamics simulations were performed first in an NPT ensemble to reach the desired pressure (0 atm) and temperature (290 K) with a timestep of 1 fs. Then the system was equilibrated in an NVE ensemble for one nanosecond. After reaching the equilibrium state, the interfacial thermal conductance was measured using the transient non-equilibrium

molecular dynamics (TNEMD) approach [18]. Two different ways: (1) fixed nanocrystal and (2) fixed solvents were used to study the interfacial thermal conductance between ligand-solvent ($h_{lig-sol}$) and ligand-nanocrystal (h_{NC-lig}), respectively. For the $h_{lig-sol}$, the nanocrystal was frozen in the original position. The temperature of the ligands was maintained at 330K while the temperature of the surrounding solvents was maintained at 290K initially using the Nose-Hoover thermostats at 0.5 ns. The thermostat acting on the ligands was switched off to allow the ligand temperature to decay to 290 K'. This temperature decay was recorded for 300 ps. For h_{NC-lig}, the solvents were fixed while the nanocrystal acted as the cold source at 290K. The MD simulation results were averaged over eight initial seeds to minimize statistical fluctuations.

The interfacial thermal conductance calculation assumed the heat transfer to have negligible internal resistance (lumped system) of the ligands, which was verified using the Biot number. The energy balance equation in the ligands is given by

$$mc_p\frac{dT_{lig}}{dt} = -Ah\left(T_{lig} - T_{NC\ or\ sol}\right) ; T_{lig}(t=0) = T_i \quad (23)$$

where $T_{lig},\ m,\ c_p$ are the temperature, mass, and specific heat capacity of the ligands. A and h refer to the surface area contact and the interfacial thermal conductance between the nanocrystal and ligands (h_{NC-lig}) or solvent and ligands ($h_{lig-sol}$). T_i is the initial temperature of ligands at 330 K. $T_{NC}\ and\ T_{sol}$ denote the nanocrystal and solvent temperature. By solving Equation 23,

$$\frac{T_{lig}(t) - T_{NC\ or\ sol}}{T_i - T_{NC\ or\ sol}} = exp\left(-\frac{t}{\tau}\right) ; \tau = \frac{mc_p}{Ah} \quad (24)$$

where τ is the ligand relaxation time. Figure S12 shows the transient temperature decay of (a) the hot ligands with the CdSe nanocrystal and (b) the hot ligands with the CCl_4 solvent. We fit these transient temperature profiles to Equation 24 to obtain the unknown h value.

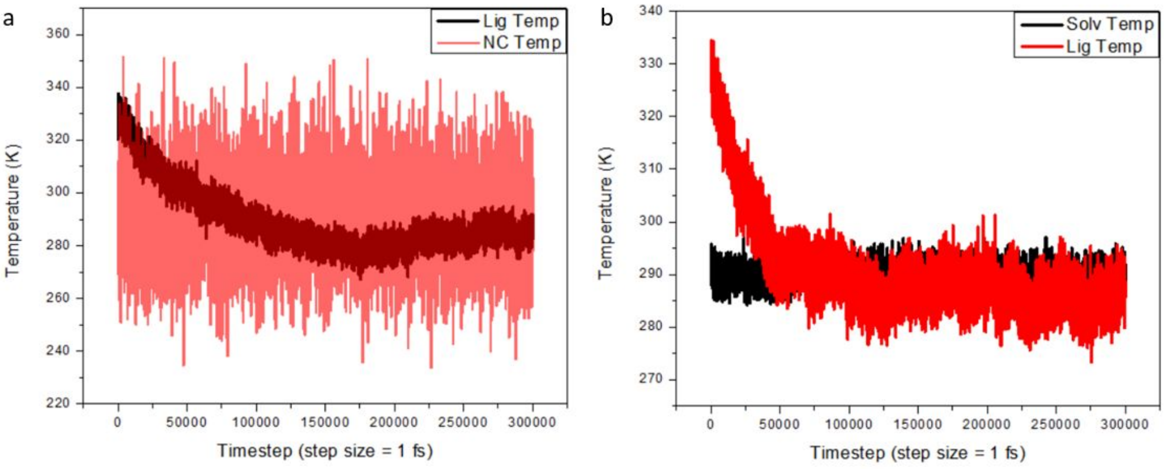

Figure S12. The temperature decay profile in the (a) hot ligands to cold nanocrystal to calculate h_{NC-lig} and the (b) hot ligands to cold solvent to calculate $h_{lig-sol}$. The initial temperature of the hot ligands and the cold sources were 330 K and 290 K.

4.3 Calculation of the number of monodentate and bidentate ligands

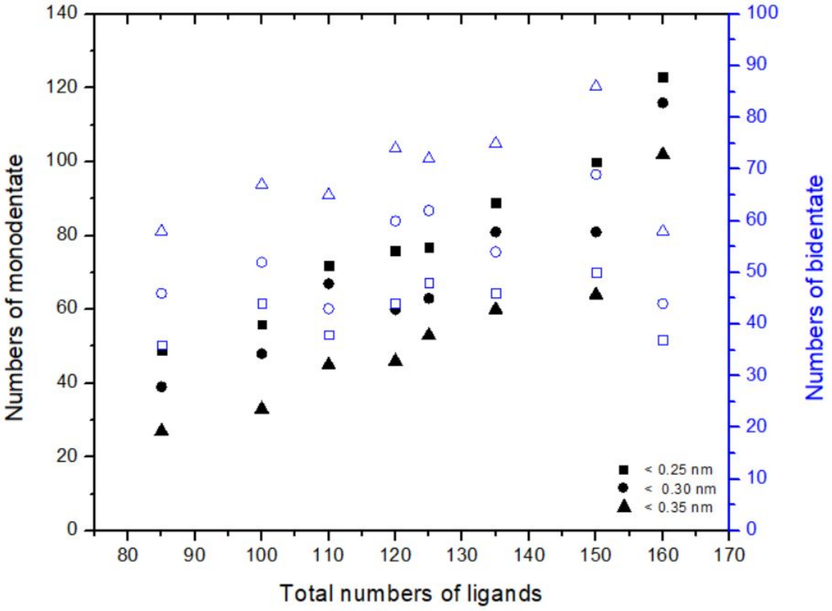

Figure S13. The calculated number of monodentate and bidentate ligands with different cut-off distances between the Cd atom and O atoms.

The Cd atom on the nanocrystal and the O atoms (i.e., the carbonyl oxygen O1 and hydroxyl oxygen O2) on the ligands influence the observed thermal conductance at the ligand-nanocrystal interface. A Python program automates the calculation of the number of

monodentate and bidentate ligands by setting a minimum distance between the Cd and O atoms. When this minimum distance between the Cd atoms and both types of O atoms in the ligand is met, this ligand is considered a bidentate ligand. Otherwise, it is a monodentate ligand. Figure S13 shows the number of monodentate and bidentate ligands as a function of the different cut-off distances between the Cd atom and O atoms. The cut-off distance of 0.30 nm between Cd atoms and O atoms is chosen as the result coincides with the results from a manual count performed on a nanocrystal with 85 ligands.

Then we calculated the radial distribution function (RDF) of the oxygen around Cd atoms to confirm the distance criterion of 3 Å is reasonable. We measured the radial distribution of the carbonyl oxygen (O1) and hydroxyl oxygen (O2) to the Cd atoms (Figure S14). The first minima are at 2.65 Å and 2.86 Å for the Cd-O1 and Cd-O2, respectively. On the other hand, the second maxima are at 3.2 Å and 4 Å. Thus, a distance criterion of 3 Å is used to differentiate between monodentate and bidentate binding as this criterion captures both the O1 and O2 types when a bidentate occurs. Moreover, in another literature[19], the authors used TIP3P, SPC, and SPCE potentials to examine the interactions between a CdSe nanocrystal and water molecules and found a similar average value of 2.80 Å between the Cd-O interactions. This similarity further supports our distance criterion.

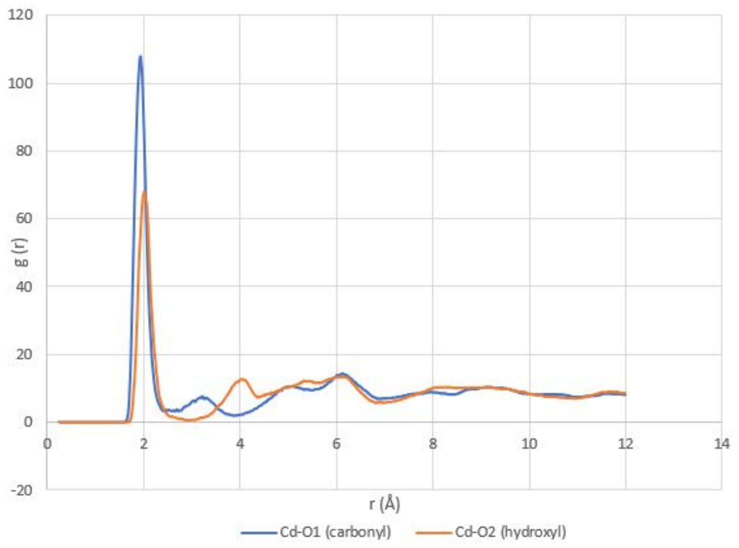

Figure S14. Radial distribution function, g(r), of Cd-O1 (carbonyl oxygen) and Cd-O2 (hydroxyl oxygen).

We also tried to bind the hydroxyl oxygens (O2) to the Cd atoms by placing them closer to the Cd atoms, without the carbonyl oxygens (O1) pointing away. However, after relaxation, the Cd atoms still bind to the O1 for the monodentate case (Figure S15). We cannot find any monodentate ligand with its O2 bound to the CdSe nanocrystal.

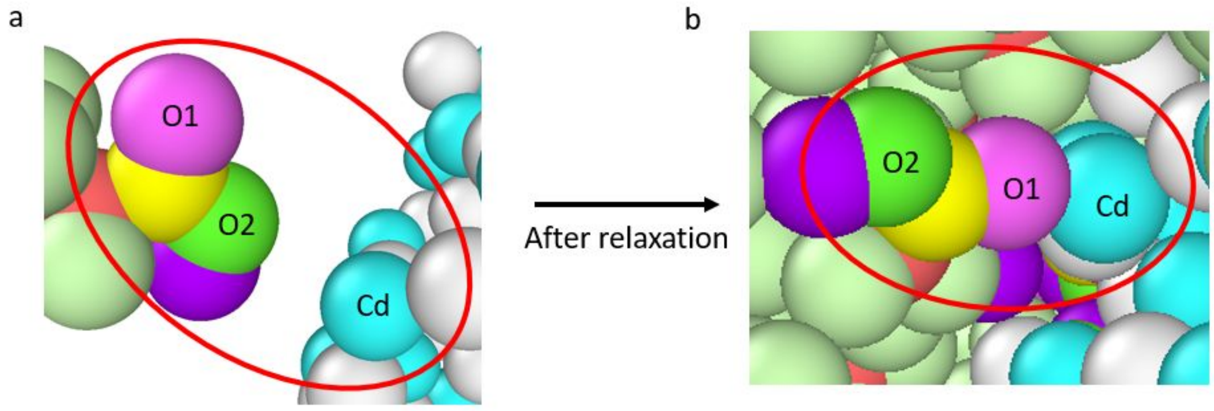

Figure S15. (a) Illustration of the hydroxyl oxygen (O2, green ball) placed facing the Cd atom (Cd, cyan ball) with the carbonyl oxygen (O1, magenta ball) tilted away from the Cd atom. (b) The carbonyl oxygen (O1, magenta ball) got bound to the Cd atom (cyan ball) after structure relaxation.

4.4 Comparison between the results with protonated and deprotonated states of ligands

For the study of thermal conductance between nanocrystal-ligand interface, both protonated and deprotonated states (oleate vs oleic acid) using MD simulations were considered. The results are shown below. For the 642 CdSe nanocrystal (324 Cd atoms and 318 Se atoms) with 85 oleic acid ligands model, a thermal conductance of 69 ± 10 MW/m^2K was obtained for the nanocrystal-ligand interface. We found that the oleate ligands tend to dislodge from the nanocrystal surface after reaching a critical number of 56 ligands. This number of the oleate ligands bound to the nanocrystal surface is still lower than the lowest grafting density in our experiments. One of the main reasons is the repulsion from the coulomb interactions between the negatively-charged oleate ligands at higher grafting density. Two different methods were attempted to increase the grafting density of oleate ligands: (1) Increase the net charge of the core by changing the charge values for both the Cd and Se atoms; (2) Delete the negatively-charged Se atoms to increase the net charge of the core.

For method (1), the number of atoms in the core was not changed (i.e., 642 CdSe with 324 Cd atoms and 318 Se atoms). The charge of the Cd atom was increased from +0.9768 to +0.9968 and the charge of the Se atom was decreased from -0.9768 to -0.9268 to create a net charge of +28.24. No appreciable change to the core diameter is seen. Here, we successfully grafted 84 oleate ligands. This model of 642 CdSe – 84 oleate ligands gives a thermal conductance of 73 ± 12 MW/m^2K.

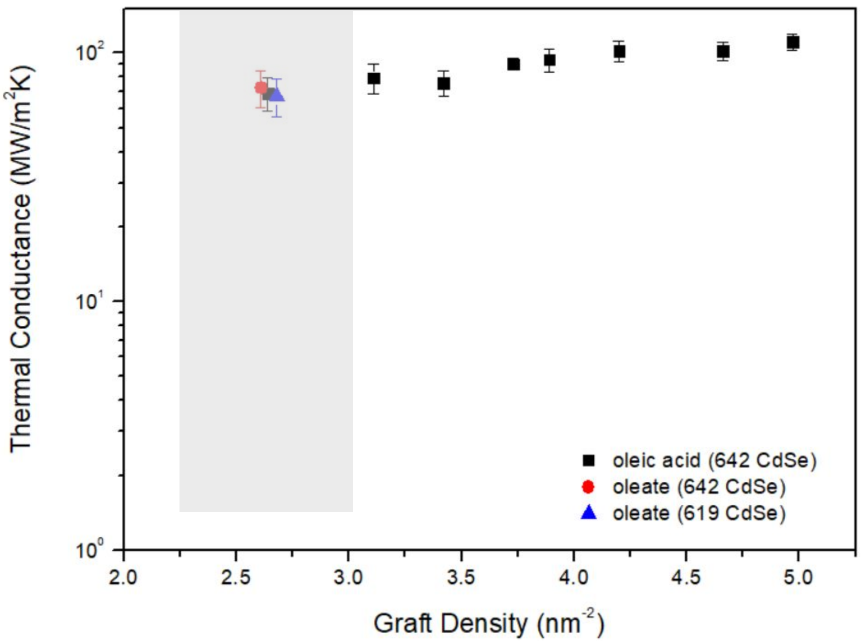

Figure S16. Thermal conductance for the three different models. Black square – original data; Red circle – keeping 642 CdSe with charge modification; Blue triangle – 619 CdSe (offset by +0.04 nm^{-2} along the x-axis for clarity).

For method (2), 23 Se atoms were removed to get a net charge of +28.33 on the core. This model of 619 CdSe (324 Cd atoms and 295 Se atoms) – 85 oleate ligands gives a thermal conductance of 67 ± 12 MW/m^2K (after taking into account a decrease of 0.06 nm in the diameter). From Figure S16, the three different models show similar thermal conductance between the nanocrystal-ligand interface at the grafting density of ~2.61 nm^{-1}. These similarities suggest that the results of the thermal conductance produced by these two types of ligands are indistinguishable within the error of molecular dynamics.

Reference:

(1) Yang, Y. A.; Wu, H.; Williams, K. R.; Cao, Y. C. Synthesis of CdSe and CdTe Nanocrystals without Precursor Injection. *Angew. Chem., Int. Ed. Engl.* **2005**, *44* (41), 6712–6715. https://doi.org/10.1002/anie.200502279.

(2) Yu, W. W.; Qu, L.; Guo, W.; Peng, X. Experimental Determination of the Extinction Coefficient of CdTe, CdSe, and CdS Nanocrystals. *Chem. Mater.* **2003**, *15* (14), 2854–2860. https://doi.org/10.1021/cm034081k.

(3) Chen, J.; Song, J. L.; Sun, X. W.; Deng, W. Q.; Jiang, C. Y.; Lei, W.; Huang, J. H.; Liu, R. S. An Oleic Acid-Capped CdSe Quantum-Dot Sensitized Solar Cell. *Appl. Phys. Lett.* **2009**, *94* (15), 153115. https://doi.org/10.1063/1.3117221.

(4) Singleton, W. S.; Ward, T. L.; Dollear, F. G. Physical Properties of Fatty Acids. I. Some Dilatometric and Thermal Properties of Stearic Acid in Two Polymorphic Forms. *J Am Oil Chem Soc* **1950**, *27* (4), 143–146. https://doi.org/10.1007/BF02634385.

(5) V.I. Grytsiv, V.N. Tomashik, Z.F. Tashik. *Inorg. Mater.* **1979**, 30.

(6) *CRC Handbook of Chemistry and Physics: A Ready-Reference Book of Chemical and Physical Data*, 92nd ed., 2011–2012.; Haynes, W. M., Lide, D. R., Eds.; CRC Press: Boca Raton, Fla., 2011.

(7) Plimpton, S. Fast Parallel Algorithms for Short-Range Molecular Dynamics. *Journal of Computational Physics* **1995**, *117* (1), 1–19. https://doi.org/10.1006/jcph.1995.1039.

(8) Luchinsky, D. G.; Hafiychuk, H.; Hafiychuk, V.; Wheeler, K. R. *Molecular Dynamics of ULTEM 9085 for 3D Manufacturing: Spectra, Thermodynamic Properties, and Shear Viscosity.*; 2018.

(9) Tamir, E.; Sidess, A.; Srebnik, S. Thermodynamic, Structural, and Mechanical Properties of Fluoropolymers from Molecular Dynamics Simulation: Comparison of Force Fields. *Chemical Engineering Science* **2019**, *205*, 332–340.

(10) O'Connor, W. E.; Warzoha, R.; Weigand, R.; Fleischer, A. S.; Wemhoff, A. P. Thermal Property Prediction and Measurement of Organic Phase Change Materials in the Liquid Phase near the Melting Point. *Applied energy* **2014**, *132*, 496–506.

(11) Ma, W.; Hong, T.; Xie, T.; Wang, F.; Luo, B.; Zhou, J.; Yang, Y.; Zhu, H.; Huang, K. Simulation and Analysis of Oleic Acid Pretreatment for Microwave-Assisted Biodiesel Production. *Processes* **2018**, *6* (9), 142.

(12) *Persson K. Materials Data on CdSe (SG:186) by Materials Project. 2014.*

(13) Moreira, N. H.; Skaf, M. S. Structural Characterization of the H2O/CCl4 Liquid Interface Using Molecular Dynamics Simulations. In *Surface and Colloid Science*; Springer Berlin Heidelberg: Berlin, Heidelberg, 2004; pp 81–85. https://doi.org/10.1007/b97086.

(14) Dodda, L. S.; Cabeza de Vaca, I.; Tirado-Rives, J.; Jorgensen, W. L. LigParGen Web Server: An Automatic OPLS-AA Parameter Generator for Organic Ligands. *Nucleic Acids Research* **2017**, *45* (W1), W331–W336. https://doi.org/10.1093/nar/gkx312.

(15) Dodda, L. S.; Vilseck, J. Z.; Tirado-Rives, J.; Jorgensen, W. L. 1.14*CM1A-LBCC: Localized Bond-Charge Corrected CM1A Charges for Condensed-Phase Simulations. *J. Phys. Chem. B* **2017**, *121* (15), 3864–3870. https://doi.org/10.1021/acs.jpcb.7b00272.

(16) Jorgensen, W. L.; Tirado-Rives, J. Potential Energy Functions for Atomic-Level Simulations of Water and Organic and Biomolecular Systems. *Proc. Natl. Acad. Sci. U.S.A.* **2005**, *102* (19), 6665–6670. https://doi.org/10.1073/pnas.0408037102.

(17) Roodbari, M.; Abbasi, M.; Arabha, S.; Gharedaghi, A.; Rajabpour, A. Interfacial Thermal Conductance between TiO2 Nanoparticle and Water: A Molecular Dynamics Study. *Journal of Molecular Liquids* **2022**, *348*, 118053. https://doi.org/10.1016/j.molliq.2021.118053.

(18) Rajabpour, A.; Seif, R.; Arabha, S.; Heyhat, M. M.; Merabia, S.; Hassanali, A. Thermal Transport at a Nanoparticle-Water Interface: A Molecular Dynamics and Continuum Modeling Study. *J. Chem. Phys.* **2019**, *150* (11), 114701. https://doi.org/10.1063/1.5084234.

(19) de Araujo, A. S.; Sonoda, M. T.; Piro, O. E.; Castellano, E. E. Development of New Cd^{2+} and Pb^{2+} Lennard-Jones Parameters for Liquid Simulations. *J. Phys. Chem. B* **2007**, *111* (9), 2219–2224. https://doi.org/10.1021/jp064835t.
